# Insights into ClpXP proteolysis: heterooligomerization and partial deactivation enhance chaperone affinity and substrate turnover in *Listeria monocytogenes* †
†Electronic supplementary information (ESI) available: Figures, tables and experimental procedures. See DOI: 10.1039/c6sc03438a



**DOI:** 10.1039/c6sc03438a

**Published:** 2016-10-28

**Authors:** Dóra Balogh, Maria Dahmen, Matthias Stahl, Marcin Poreba, Malte Gersch, Marcin Drag, Stephan A. Sieber

**Affiliations:** a Center for Integrated Protein Science at the Department of Chemistry , Technische Universität München , Lichtenbergstraße 4 , Garching bei München , D-85747 , Germany . Email: stephan.sieber@mytum.de; b Department of Bioorganic Chemistry , Faculty of Chemistry , Wrocław University of Technology , Wybrzeże Wyspiańskiego 27 , 50-370 Wrocław , Poland

## Abstract

Caseinolytic proteases (ClpP) are important for recognition and controlled degradation of damaged proteins. While the majority of bacterial organisms utilize only a single ClpP, *Listeria monocytogenes* expresses two isoforms (LmClpP1 and LmClpP2). LmClpPs assemble into either a LmClpP2 homocomplex or a LmClpP1/2 heterooligomeric complex. The heterocomplex in association with the chaperone ClpX, exhibits a boost in proteolytic activity for unknown reasons. Here, we use a combined chemical and biochemical strategy to unravel two activation principles of LmClpPs. First, determination of apparent affinity constants revealed a 7-fold elevated binding affinity between the LmClpP1/2 heterocomplex and ClpX, compared to homooligomeric LmClpP2. This tighter interaction favors the formation of the proteolytically active complex between LmClpX and LmClpP1/2 and thereby accelerating the overall turnover. Second, screening a diverse library of fluorescent labeled peptides and proteins with various ClpP mutants allowed the individual analysis of substrate preferences for both isoforms within the heterocomplex. In addition to Leu and Met, LmClpP2 preferred a long aliphatic chain (2-Aoc) in the P1 position for cleavage. Strikingly, design and synthesis of a corresponding 2-Aoc chloromethyl ketone inhibitor resulted in stimulation of proteolysis by 160% when LmClpP2 was partially alkylated on 20% of the active sites. Determination of apparent affinity constants also revealed an elevated complex stability between partially modified LmClpP2 and the cognate chaperone LmClpX. Thus, the stimulation of proteolysis through enhanced binding to the chaperone seems to be a characteristic feature of LmClpPs.

## Abstract 2

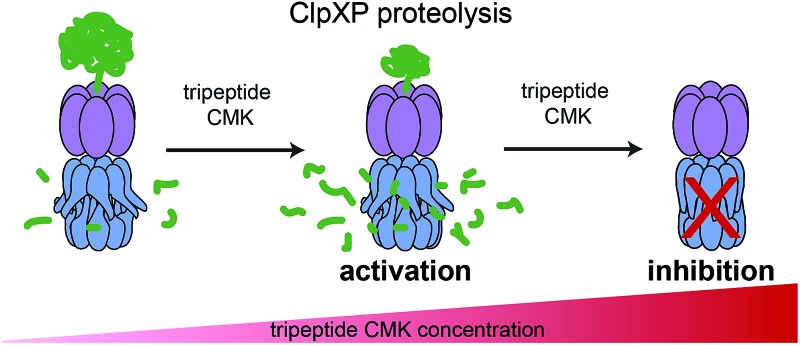
Caseinolytic protease from *Listeria* exploits two paths of proteolytic stimulation: heterooligomerization and partial inhibitor binding both enhance ClpX chaperone affinity.

## Introduction

ATP-dependent proteolysis represents an important mechanism for removal of misfolded or ribosome-stalled proteins under stress conditions. In prokaryotes AAA+ chaperones (such as ClpX, ClpA and ClpC) recognize and unfold substrate proteins by ATP consumption and direct the linear peptide chain into a proteolytic barrel of caseinolytic protease P (ClpP).^1–4^ ClpP is a transient tetradecameric serine hydrolase composed of two heptameric rings that are stacked face-to-face. Each subunit carries an active Ser-His-Asp catalytic triad that is essential for activity.^5,6^ ClpP by itself lacks proteolytic activity but is able to digest small peptides that diffuse into the barrel *via* axial pores.^7,8^ The first specific inhibitors reported for ClpP include the beta-lactones, which exhibit an irreversible mode of action and, depending on their chemical structure, can cause either retention of the tetradecameric state or deoligomerization of ClpP.^9,10^ More detailed insights into inhibitor-mediated complex disassembly were provided through a new generation of covalent phenyl esters and relevant modeling studies. These studies suggested that steric clash of the inhibitor within the active site triad triggers a rearrangement at the heptamer interface, causing dissociation of the ClpP tetradecamer into two heptameric rings.^11^ Recently, the first reversible ClpP inhibitors were reported, which distort the active site catalytic triad through structural rearrangements.^12^ However, this inactive state of the ClpP peptidase could be reversed through formation of the ClpXP complex, highlighting the power of conformational control within this dynamic system. ClpXP interaction is mainly mediated by ClpX-loops which bind into hydrophobic clefts located at the ClpP axial surface.^13^

Interestingly, some bacterial strains such as *Mycobacterium tuberculosis* and *Listeria monocytogenes* encode two ClpP isoforms (ClpP1 and ClpP2).^14–18^ While *L. monocytogenes* ClpP2 (LmClpP2) resembles related enzymes in other bacteria such as *Escherichia coli* and *Staphylococcus aureus*, LmClpP1 shares only 44% sequence identity with *E. coli* ClpP, forms predominantly inactive heptamers, lacks a conserved N-terminal chaperone binding motif and exhibits a truncated catalytic triad in which Asp172 is replaced by an Asn residue.^15^ Mutational studies and in-depth X-ray crystallographic analysis revealed that this Asn residue is responsible for a conformational selection of the inactive heptameric state.^15^ Accordingly, mutation of this Asn to an Asp induced tetradecamer formation and increased catalytic activity of LmClpP1.^15^

Negative stain EM images of mixed LmClpP1 and LmClpP2 indicated the formation of heterocomplexes composed of two homoheptameric rings.^14^ Importantly, it was found that LmClpP1 is only active when complexed with LmClpP2, which forces LmClpP1 into an active conformational state.^15^ The molecular reason for this surprising finding could be explained by a crystal structure of the LmClpP1/2 heterocomplex.^16^ All active sites within the heterocomplex, including Asn of LmClpP1, were aligned in an active conformation, which demonstrated that heterocomplex formation regulates LmClpP1 activity. While the heterocomplex was less active in peptidase assays, a 10-fold increase in proteolytic activity over the homocomplex was observed when in the presence of LmClpX. From a functional perspective, this implies that the cell produces a hyperactive enzyme during stress conditions, when misfolded proteins must be removed efficiently. Indeed, quantitative real-time PCR showed increased *clpP1* and *clpP2* expression under heat stress.^16^ So far, no systematic analysis of LmClpP1 and LmClpP2 peptidolytic and proteolytic cleavage specificities has been performed, leaving their role in substrate recognition unresolved. Structural studies and beta-lactone inhibitor screenings showed subtle differences in the P1 binding pockets and revealed a preference of LmClpP2 for medium to long aliphatic and aromatic side chains. LmClpP1, on the other hand, did not bind inhibitors specifically and only bound the natural product vibralactone.^14,15^

Interestingly, ClpP1 and ClpP2 from *M. tuberculosis* (MtClpP1 and MtClpP2) also assemble into a similar heterocomplex.^17–19^ However, many mechanistic and functional differences compared to the *L. monocytogenes* heterocomplex have been reported. For example, MtClpP1 and MtClpP2 are both tetradecamers, which show only weak proteolytic activity on their own in the presence of chaperones.^17^ Peptidolytic activity is only enabled through MtClpP1/2 heterocomplex formation and requires the presence of activating peptides.^17,19,20^ Proteolytic substrate turnover is facilitated in association with the chaperones MtClpX or MtClpC1.^17,21^ Moreover, peptide substrate screenings with MtClpP1 revealed a cleavage preference after Met, Leu, Phe and Ala residues, while MtClpP2 was largely inactive.^22^ Although structural information remains elusive, mutational studies within the hydrophobic clefts of MtClpPs showed that chaperones only bind the heterocomplex *via* MtClpP2.^23^ The stoichiometry of the *L. monocytogenes* complexes remains unknown. However, as tetradecameric LmClpP1(N172D) is inactive in proteolysis assays with LmClpX, the LmClpP1/2 heterocomplex does not seem to bind LmClpX *via* the ClpP1 ring.^16^

Here, we utilize peptide libraries to identify LmClpP1 and LmClpP2 cleavage specificities. While these preferences were largely abrogated in the proteolytic complexes, both isoforms retained a certain degree of individual specificity in protein digests. Biochemical analyses were used to dissect the multiple steps of proteolysis and revealed that the elevated activity of the heterocomplex stems from a 7-fold increased binding affinity for the LmClpX chaperone. Surprisingly, stimulation of proteolysis was also observed when a customized chloromethyl ketone-based (CMK) inhibitor partially modified the active sites of homotetradecameric LmClpP2. A closer mechanistic inspection of this inhibition mode revealed an increased affinity for the chaperone LmClpX to be the fundamental activation principle.

## Results and discussion

### LmClpP1 and LmClpP2 exhibit cleavage site specificity in peptidase screenings

**Fig. 1:**
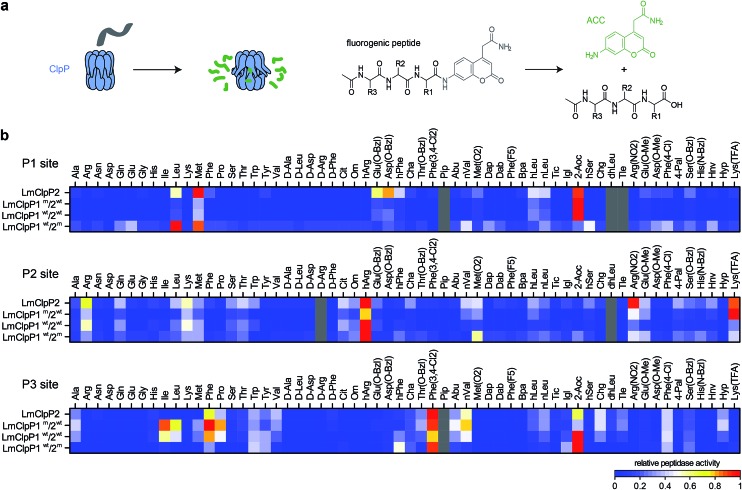
Peptide library screenings. (a) Principle of ClpP peptidase assay: after cleavage of an ACC-tagged tripeptide by ClpP an increase in fluorescence is measured (please refer to ESI† for the structures of the R1, R2 and R3 residues). (b) Cleavage specificity of LmClpP variants in peptidase assays represented as a heatmap. Each row is normalized to the lowest (0) and to the highest (1) activity. Substrates in gray and peptides containing Cys could not be obtained. Please refer to the ESI† for the structures of the substrates, for the nomenclature of non-natural amino acids and for the absolute values and errors of the peptidase activity. Peptidase activities were measured in triplicates.

A structural overlay of LmClpP1 and LmClpP2 revealed subtle differences in their S1 pockets.^16^ Specifically, LmClpP1 exhibited a smaller and more defined cleft, restricting accessibility to smaller substrate side chains, while LmClpP2 resembles ClpPs from other organisms, *e.g. E. coli*, and provides access to larger substituents.^24^ In light of this divergence, we tested a previously established synthetic library of 172 fluorogenic 7-amino-4-carbamoylmethylcoumarin (ACC)-tagged peptide substrates containing various natural and unnatural amino acid substituents at the P1, P2 and P3 sites (Fig. 1a).^25^ Cys was omitted from the substrate library since this amino acid is susceptible to oxidation under synthesis conditions, storage and its use in kinetic assays. The library was screened against LmClpP2, wild type LmClpP1/2 (LmClpP1^wt^/2^wt^) as well as the active site mutants LmClpP1/2(S98A) (LmClpP1^wt^/2^m^) and LmClpP1(S98A)/2 (LmClpP1^m^/2^wt^) in order to unravel cleavage preferences of the individual heterocomplexes (Fig. 1b). Interestingly, in the mutated heterocomplexes, LmClpP1 favored small Leu and Met residues while LmClpP2 was capable of cleaving substrates containing a large 2-aminooctanoic acid (2-Aoc) group at the P1 position. This overall specificity reflects the available space within the S1 pockets and corresponds well with previous data in which beta-lactone inhibitors with large, hydrophobic chains effectively inhibited LmClpP2, but not LmClpP1.^15^ A cleavage preference for 2-Aoc was also previously shown for *S. aureus* SaClpP, which shares 78% sequence identity with LmClpP2, suggesting an evolutionarily conserved function of these highly homologous ClpPs (Fig. S1†).^25^ Similarly, LmClpP1^wt^/2^wt^ preferred 2-Aoc-containing substrates, as a result of the increased peptidolytic activity of LmClpP2 in the heterocomplex and its influence on the cleavage pattern.

Comparison of P2 and P3 libraries revealed a less stringent specificity and identified some additional preferences and similarities of cleavage sites (Fig. 1a). For example, homoarginine (hArg) was well tolerated at the P2 position and 3,4-dichlorophenylalanine at the P3 position by both LmClpP variants. d-Amino acids were not processed, suggesting a high degree of stereochemical discrimination by LmClpP. For example, Leu at the P1 position was readily turned over in its l-configuration, but the corresponding d-stereoisomer could not be processed by any of the constructs. As the substrate specificity extends beyond the scope of natural amino acids, it is possible that chaperone-independent hydrolysis of peptidic, cellular metabolites could be an additional function of LmClpP1/2.

### Cleavage specificity in protease assays

**Fig. 2:**
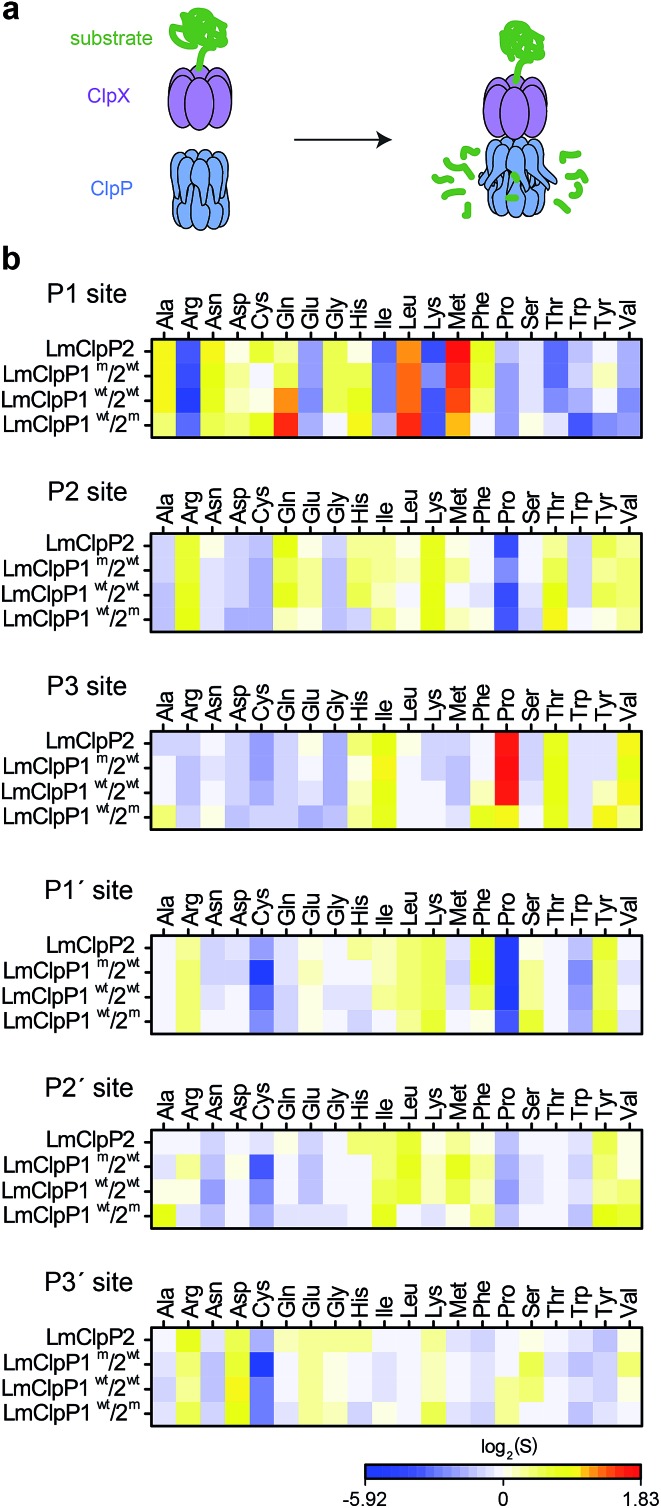
Cleavage specificity in a protease assay. (a) Principle of ClpP protease assay: SsrA-tagged substrate proteins are digested by the ClpXP complex and the produced peptides are analyzed by MS/MS. (b) Cleavage specificity of LmClpP variants in protease assays represented as a heatmap. ClpXP digests of substrate proteins were analyzed by Protein|Clpper.^25^ log_2_(*S*) > 0 means that cleavage occurred more often than expected from the amino acid composition of the substrate proteins, log_2_(*S*) < 0 represents less frequent cleavage than random cleavage. Data represent two independent experiments with five different substrate proteins.

Having identified substrate preferences of LmClpP1 and LmClpP2 at the peptide level, we investigated if this specificity holds true for cleavage of proteins as well. Therefore, two cognate *L. monocytogenes* ClpP substrate proteins, serine hydroxymethyltransferase (LmGlyA) and nicotinate phosphoribosyltransferase (LmPncB), were fused to a ClpX SsrA recognition tag and the purified recombinant proteins were subjected to LmClpXP protease assays (Fig. 2a). SsrA-tagged green fluorescent protein (GFP), RNA polymerase sigma factor (EcRpoS) and NAD-specific glutamate dehydrogenase (SaGudB), substrates previously used in EcClpXP and SaClpXP protease assays,^25^ were also included to broaden the substrate scope of the study. We tested each of the four LmClpP2, LmClpP1^wt^/2^wt^, LmClpP1^wt^/2^m^ and LmClpP1^m^/2^wt^ constructs and analyzed protein digests *via* high-resolution tandem mass-spectrometry (MS/MS) coupled to a nano-HPLC using established procedures.^25^ Peptide fragments were sequenced and analyzed *via* the Protein|Clpper software (; http://www.oc2.ch.tum.de). The algorithm calculates log_2_ scores *S* by dividing the occurrence of an amino acid A at cleavage site position P by the natural occurrence of this amino acid in the respective protein. Thus, log_2_(*S*) values larger than 0 indicate that the respective residue is enriched at a given position, whereas log_2_(*S*) values smaller than 0 reflect depletion.^25^ Overall, a sufficient sequence coverage comprising 4668 unique peptides and 11 508 peptide-to-spectrum matches were obtained, which led to the analysis of 22 925 cleavage reactions (3221 for LmClpP2, 4647 for LmClpP1^wt^/2^wt^, 8618 for LmClpP1^wt^/2^m^ and 6439 for LmClpP1^m^/2^wt^) (Fig. 2b). Although less pronounced compared to peptidase data, a distinct cleavage pattern was observed at the P1 site with a preference for Leu and Met by both LmClpP isoforms, which matches previous ClpXP analyses.^22,25^ Strikingly, a difference in specificity was observed for LmClpP1, which also preferred cleavage after Gln. This preference was less prominent in the peptidase assays and is unique to this proteolytic complex amidst other ClpXPs investigated so far. In contrast to the peptide substrate library screening, only a few amino acids (Arg, Lys, Trp for all enzymes and Ile and Thr for LmClp2) were strongly depleted in the protease screening. In addition, Protein|Clpper analysis of the digests showed much lower overall specificity at the P2, P3 and prime sites than at the P1 site. Nevertheless, Pro appears to be a crucial cleavage blocker if present at P2 or P1′ sites, which is likely attributable to its rigidity and locking the protein backbone in a kinked conformation. However, Pro at P3 increases the probability of being processed in the case of LmClpP2.

### Identification of LmClpP1/LmClpP2 peptidase and protease inhibitors

**Fig. 3:**
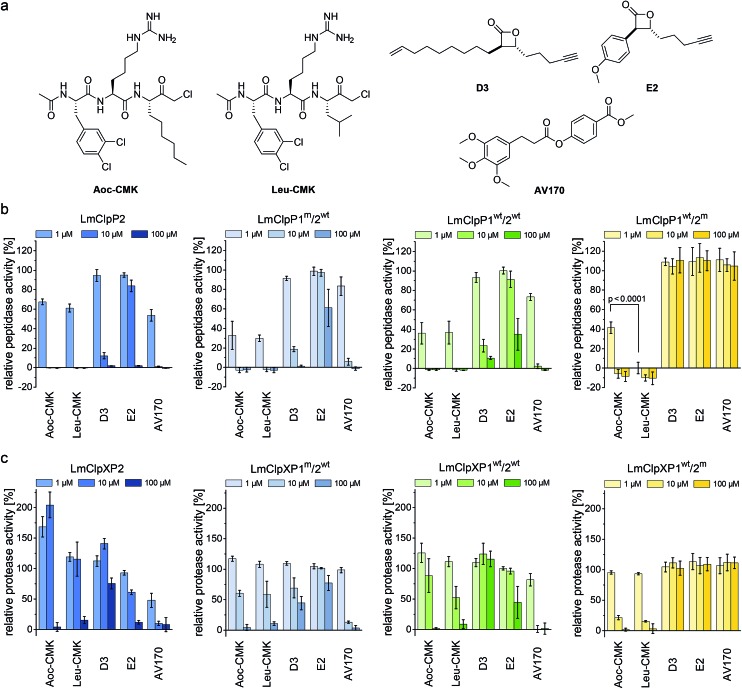
Screening of LmClpP inhibitors. (a) Structures of the inhibitors used in this study. (b) Peptidase assay (1 μM LmClpP and 200 μM Ac-Ala-hArg-Leu-ACC substrate). (c) Protease assay (0.2 μM LmClpP_14_, 0.4 μM LmClpX_6_ and 0.4 μM eGFP-SsrA). Three different inhibitor concentrations were tested. Data are normalized to the DMSO control as 100%. Datasets represent at least two independent experiments which were measured in triplicate (mean ± sd). *p*-Value was determined by Student's *t*-test.

Previously reported beta-lactones^9^ did not inhibit LmClpP1, thus failing to lend any insights into the heterocomplex mechanism.^15^ However, with the peptide screening data in hand, we were able to design customized LmClpP inhibitors in order to analyze the role of each isomers in more detail. A tripeptide scaffold with a C-terminal chloromethyl ketone (CMK) group was used to irreversibly bind the active site. Although peptide–CMK inhibitors are less selective and exhibit limited cell-permeability for *in situ* applications, the main advantages of these compounds are their stability upon enzyme active site binding as well as their customizability. We thus synthesized two inhibitors that incorporated the best residues from the P1, P2 and P3 library screening. Hence, 2-Aoc and Leu were selected for P1, while P2 and P3 sites contained hArg and 3,4-dichlorophenylalanine, respectively, yielding **Leu-CMK** and **Aoc-CMK** inhibitors (Fig. 3a). The synthesis of these compounds was performed in a similar manner as described by Kato *et al.* ^26^ In brief, Ac-Phe(3,4-Cl_2_)-hArg-COOH dipeptide was synthesized on 2-chlorotrityl chloride resin and coupled with NH_2_-Leu-CMK (or NH_2_-2-Aoc-CMK). In addition, we included **E2** and **D3** in our study as representative beta-lactone inhibitors, as well as **AV170**, a recently introduced ClpP inhibitor with an electrophilic phenyl ester moiety (Fig. 3a).^11^

The peptidolytic activity of LmClpP2, LmClpP1^wt^/2^wt^, LmClpP1^wt^/2^m^, LmClpP1^m^/2^wt^ and LmClpP1(N172D) was measured by the hydrolysis of Ac-Ala-hArg-Leu-ACC, a substrate that showed optimal turnover by both isomers. Inhibitors were used in three concentrations, 100, 10 and 1 μM to estimate their potency (Fig. 3b and S2†). As all complexes deviate in their peptidolytic rates, substrate turnover in the absence of inhibitor was normalized to 100% activity. **AV170** and both CMK inhibitors were the most effective against LmClpP2, followed by **D3** and **E2**. A similar profile was observed for heterooligomeric LmClpP1^wt^/2^wt^ and LmClpP1^m^/2^wt^, suggesting that inhibition of LmClpP2 alone is sufficient to affect the overall complex activity. In contrast, the inhibition pattern for LmClpP1 in LmClpP1^wt^/2^m^ and LmClpP1(N172D) complexes were quite different. Here, only the CMKs showed any inhibitory effect, and **Leu-CMK** exhibited significantly higher potency compared to the 2-Aoc analog. **Leu-CMK** completely abolished LmClpP1 activity in the heterocomplex at 1 μM, reflecting its preference for small residues at the P1 site. With the first potent LmClpP1 inhibitors in hand, we commenced with mechanistic studies of LmClpXP proteolysis.

### Partial inhibition of homooligomeric LmClpP2 stimulates proteolysis

**Fig. 4:**
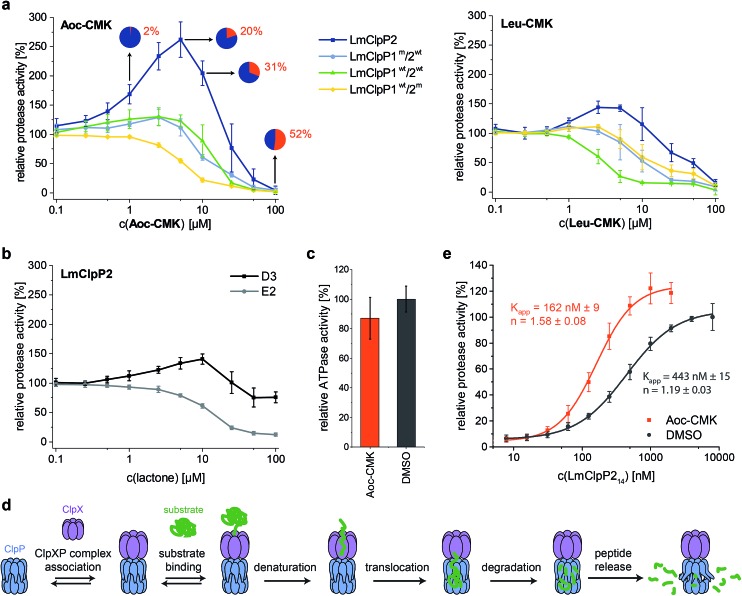
Activation of the ClpXP proteolysis by small molecules. (a) Protease activity of LmClpP variants with **Aoc-CMK** and **Leu-CMK**. Pie diagrams illustrate the degree of modification of LmClpP2 with **Aoc-CMK**, determined by intact protein mass spectrometry (means from triplicate experiments are shown). (b) Protease activity of the LmClpP2 homocomplex with lactones **D3** and **E2**. (c) ATPase activity of LmClpX_6_ (0.2 μM) in the presence of LmClpP2_14_ (0.1 μM) and **Aoc-CMK** (4.25 μM) with 20 mM ATP. Data represent three independent experiments which were measured in quadruplicate (mean ± sd). (d) Kinetic scheme for protein degradation by ClpXP (parts of this figure have been adapted from Kim *et al.* ^27^). (e) Protease activity of LmClpP (varying concentrations of LmClpP_14_) with and without **Aoc-CMK** (in 25-fold excess to LmClpP2_14_). Curves were fit to the Hill equation (*y* = *V*_min_ + (*V*_max_ – *V*_min_)*x* ^*n*^/(*K*_app_^*n*^ + *x* ^*n*^)). Protease activity data represent at least two independent experiments which were measured in triplicate (mean ± sd).

LmClpP2, LmClpP1^wt^/2^wt^, LmClpP1^wt^/2^m^ and LmClpP1^m^/2^wt^ were reconstituted with LmClpX and proteolysis of GFP-SsrA was monitored by a decrease in fluorescence signal according to previously established protocols.^27^ Inhibitors were added at three concentrations (100, 10 and 1 μM) to estimate their effects on enzymatic activity. Phenyl ester **AV170** turned out to be the most effective inhibitor against each complex, except LmClpXP1^wt^/2^m^, highlighting the restricted binding site preferences of the LmClpP1 isoform (Fig. 3c). Similarly, none of the beta-lactones were able to inhibit LmClpP1, although the newly designed CMKs showed pronounced effects on both isoforms. The compounds reduced proteolysis of all three heterocomplex constructs in a concentration-dependent manner, but, surprisingly, **Aoc-CMK** enhanced GFP degradation by the LmClpXP2 homocomplex. A similar, but less pronounced, proteolytic stimulation of LmClpXP2 was observed with aliphatic beta-lactone **D3**. To investigate this unexpected finding in more detail, proteolytic assays with all complexes were performed at incremental inhibitor concentration steps (Fig. 4a). Interestingly, 5 μM **Aoc-CMK** resulted in the strongest activation of LmClpXP2 proteolysis (162%) while higher concentrations decreased, and finally abolished, complex activity at 50 μM. Intact-protein MS analysis of the most activated species revealed 20% LmClpXP2 complex occupancy of the inhibitor. This suggests that incomplete inhibitor binding to the LmClpP2 tetradecamer stimulates proteolysis in association with LmClpX, but not peptidolysis, when in the absence of chaperone (see results above). The degree of modification reached 52% at 100 μM, which was sufficient to fully abolish proteolytic activity (Fig. 4a). As CMKs are general ClpP inhibitors, we tested the effect of partial **Aoc-CMK** activation with the *S. aureus* SaClpXP system and obtained 26% stimulation, highlighting that this intriguing phenomenon is less pronounced in other homologs and may thus be a specific feature of LmClpP2 (Fig. S3†).

Importantly, partial binding alone cannot explain the proteolytic enhancement since aromatic lactone **E2** inhibited LmClpXP2 without significant activation, while **D3** showed 41% activation (Fig. 4b). In addition, all three heterocomplex constructs revealed only marginal to no enhancement of turnover with **Aoc-CMK**, suggesting that homotetradecameric LmClpP2 is required to trigger this effect. Similarly, **Leu-CMK** stimulated LmClpXP2 to a much lesser extent than **Aoc-CMK**, highlighting that both the intrinsic reactivity as well as the compound structure are crucial for the activity. We thus commenced with in-depth analysis of the mechanistic requirements responsible for these effects.

### Alkylation of LmClpP2 triggers LmClpX binding

It is known that LmClpP1/2 heterooligomerization, in combination with LmClpX chaperone binding, enhances proteolysis.^16^ Strikingly, we observe here an additional activation mechanism, predominantly for the homocomplex, which raises the question of whether there is a common underlying mechanism. We set out to answer this question by studying the homo- and heterocomplexes with a suite of biochemical and analytical methods. For these studies, we selected **Aoc-CMK** as a suitable tool compound due to its strong stimulatory effect and irreversible mode of alkylation.

First, we focused on the homocomplex and addressed the nature of its activation by studying its conformational stability. Select lactones, including **E2** and phenyl esters such as **AV170**, are known to destabilize tetradecameric ClpP and to induce its dissociation into inactive heptamers.^10,11^ In fact, deoligomerization of LmClpP2 could be observed upon **E2** binding, while the tetradecameric complex was retained with both the CMKs and **D3** (Fig. S4†). This demonstrates a principle difference in the binding mode, in which those molecules that disrupt the oligomeric state thereby inactivate LmClpP, while those that only partially modify LmClpP and retain the tetradecameric state stimulate LmClpP.

Second, as partial inhibition does not trigger peptidolysis but proteolysis, we investigated the role of the associated LmClpX chaperone. ClpX recognizes tagged substrates and catalyzes their ATP-dependent unfolding, which represents the rate-determining step of proteolysis.^27^ Thus, LmClpX ATPase activity was determined in the presence of partially **Aoc-CMK**-inhibited LmClpP2. No increase in ATP turnover could be observed, suggesting that ATP hydrolysis occurs independently of the association with **Aoc-CMK**-modified LmClpP (Fig. 4c).

Third, LmClpXP2 assembly is in equilibrium with individual LmClpP2 and LmClpX complexes. This equilibrium precedes all subsequent steps required for protein turnover, including ClpX-mediated substrate binding, unfolding and translocation into the proteolytic chamber (Fig. 4d). A shift in equilibrium towards the proteolytically-active LmClpXP2 complex could therefore significantly enhance overall activity. We thus elucidated the direct interaction between LmClpP2 and LmClpX in proteolytic assays, in which the concentration of LmClpP2 was systematically varied and an apparent affinity constant was calculated. Importantly, while the *K*_app_ of LmClpXP2 with unmodified LmClpP2 was 443 nM, the *K*_app_ for partially **Aoc-CMK**-modified LmClpP2 dropped to 162 nM. This increase in affinity through partial inhibitor binding to ClpP reveals an intriguing mechanism of activation (Fig. 4e). A similar enhancement in ClpX affinity was previously observed for *E. coli* ClpP fully inactivated by diisopropyl fluorophosphonate (DFP) which contributed to a model of functional communication between ClpX and ClpP during substrate processing.^28^ Here, we show that the mechanism of proteolytic stimulation extends beyond complete active site binding and is largely affected by the degree of modification, inhibitor structure as well as the ClpP isoform investigated.

### Heterooligomeric LmClpP1/2 is intrinsically stimulated by enhanced LmClpX binding

Proteolytic studies of the three heterocomplexes did not reveal pronounced activation upon partial modification with **Aoc-CMK**. However, it should be noted that all heterocomplex constructs, including those that contain an active-site mutated ring, are about 10-fold more active compared to the LmClpXP2 homocomplex for yet unknown reasons.^16^ An effect on LmClpX ATPase activity by heterooligomerization has already been excluded in previous studies.^16^ In order to understand the basis for the enhanced activity, we dissected the mechanism and investigated key steps relevant for proteolysis.

**Fig. 5:**
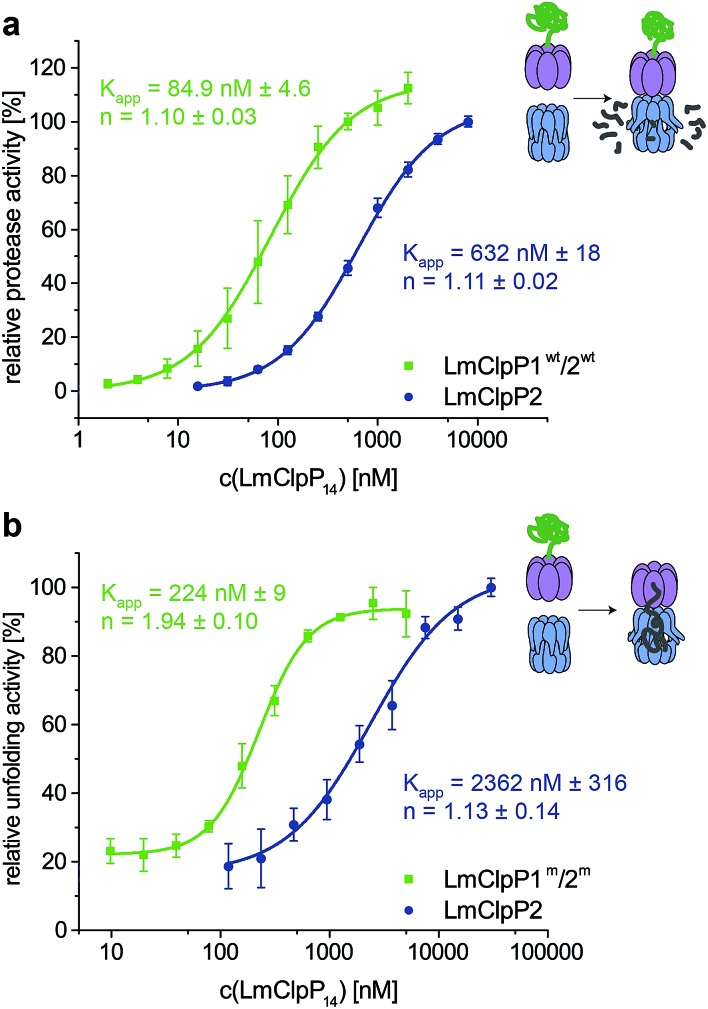
Stimulation of ClpXP activity by hetero-oligomerization. (a) Protease activity of hetero- and homocomplex of LmClpP (varying concentrations of LmClpP_14_). The data set represents three independent experiments which were measured in triplicate (mean ± sd). (b) Unfolding activity of hetero- and homocomplex of LmClpXP(S98A) (0.4 μM LmClpX_6_, varying concentrations of LmClpP_14_) in presence of GFP-LmSsrA (0.125 μM). The data set represents two independent experiments which were measured in triplicate (mean ± sd).

Inspired by the results of partial **Aoc-CMK** inhibition, we focused on the interaction between LmClpP1/2 and LmClpX, and determined their affinity. Proteolytic assays were performed with varying LmClpP1/2 concentrations, and apparent affinity constants were calculated as outlined above (Fig. 5a). Importantly, the *K*_app_ for LmClpXP1/2 was 85 nM and thus about 7-fold lower compared to the LmClpXP2 homocomplex. These results suggest a strong shift in the equilibrium of complex formation to the LmClpXP1/2 form, thereby enhancing substrate turnover.

To further prove this theory, we determined the *K*_app_ for protein unfolding, which is the rate-limiting step before proteolysis.^27^ To focus on LmClpX activity, we utilized LmClpP2 and LmClpP1/2 containing Ser98 to Ala mutations. While catalytically inactive, these mutant proteins are still able to bind LmClpX and facilitate the translocation of the linear peptide chain into the barrel. Systematic variation of LmClpP concentrations revealed *K*_app_ values of 2362 nM and 224 nM for homo- and heterocomplexes, respectively (Fig. 5b). In agreement with the proteolysis data, these results indicate that LmClpX and LmClpP1/2 form the functional complex with higher affinity at the rate-limiting, substrate-unfolding step, thereby enhancing the overall activity.

Although these studies provide a mechanistic basis for understanding the elevated proteolytic activity, the nature of this high-affinity interaction, as well as how the heterocomplex or partially-inhibited homocomplex facilitate tighter LmClpX binding, remain to be explored. No significant structural differences between the LmClpP2 homocomplex and heterocomplex with respect to LmClpP1 were observed.^16^ However, the N-terminal regions, which are crucial for chaperone interaction, could not be characterized with high resolution so far. In order to unravel more general aspects of heterocomplex assembly, analysis of its quaternary structural organization would be required.

## Conclusion

Protein degradation is tightly controlled by the cell in order to prevent unwanted proteolytic damage. Thus, major proteases such as ClpP and DegP are only activated for proteolysis in association with cognate chaperones and oligomerization, respectively.^29^ The chaperones act as gatekeepers and bind to conserved hydrophobic pockets on the apical sites of ClpP^30–32^ in order to initiate protein degradation by substrate recognition and unfolding. The mechanism of activation was more closely investigated with acyldepsipeptides (ADEPs),^30^ which are small-molecule chaperone mimics that bind to the same hydrophobic pockets and thereby, like ClpX, induce pore opening and conformational changes of ClpP to the activated state.^33^ Thus, binding to chaperones, or mimics thereof,^34^ represents the first known method of ClpP activation. Surprisingly, we identify here additional mechanisms of activation of LmClpPs, based on their affinity for chaperones. Key to this analysis was the fact that ClpP and ClpX are in a dynamic equilibrium for the formation of the proteolytically-competent ClpXP complex, which is the critical first step for proteolysis (Fig. 4d).^27^

We initiated our studies by inspecting both LmClpP isoforms and discovered that the LmClpP2 peptidase, similar to its *S. aureus* homolog,^25^ prefers substrates with a long aliphatic side chain at the P1 site. As ClpP exhibits hydrolytic activity of small substrates that access the proteolytic chamber by diffusion, it is possible that it may have specialized functions beyond the cleavage of proteins. Although the preference for aliphatic amino acids such as Leu and Met, similar to other ClpPs, was retained in proteolytic studies, the overall selectivity observed for peptidase activity was largely abrogated.^22,25^ However, a striking feature of LmClpP1 is its additional preference for Gln at the P1 site. An additional feature of LmClpP1/2 is the accelerated proteolytic rate when associated with LmClpX. We show that the reason for this second principle of ClpP activation is a higher affinity between LmClpP1/2 and LmClpX, which shifts the equilibrium to the active LmClpXP1/2 heterocomplex. As the apical sites of LmClpP1 and LmClpP2 do not significantly change in the homo- and heterocomplexes, structural differences in the loop regions, which could not be resolved so far, may be responsible for a tighter interaction. Importantly, LmClpP2 is prone to a third mechanism of activation based on partial active site binding by irreversible inhibitors. While modification of 20% of all catalytic serines by a CMK inhibitor increased proteolysis by 160%, alkylation of approximately 50% of the sites resulted in full inhibition. Again, the reason for this proteolytic stimulation was an increase in binding affinity of the chaperone for partially CMK-modified LmClpP2, which shifted the equilibrium to the active proteolytic complex. Thus, modulating the affinity between ClpP and ClpX seems to be a unifying principle for proteolytic fine-tuning. Interestingly, the activation heavily depended on the inhibitor used. While **Aoc-CMK** induced the strongest effect, a CMK inhibitor containing Leu at the P1 site was only marginally stimulatory, suggesting that binding of long aliphatic chains into the S1 pocket could be a crucial parameter. Similarly, the long aliphatic beta-lactone inhibitor **D3** also stimulated proteolysis, although to a much lesser extent, which suggests that this effect is more general and can also be observed by lactone active-site acylation in addition to CMK alkylation. On the contrary, aromatically-decorated lactone **E2**, a known disruptor of the ClpP tetradecamer,^10^ inhibited turnover, which emphasizes the importance of the ligand introduced into the S1 pocket. While a slight activation by **Aoc-CMK** was also observed for SaClpP, the more pronounced effects in LmClpP2 suggest a more functionally relevant role in this system. Based on this data, the LmClpP2 active site and substrate pocket seem to control the fate of proteolytic activity. Partial binding of long aliphatic ligands or heterooligomerization with LmClpP1 induce proteolysis *via* tighter binding to ClpX, which may be necessary under certain stress conditions. For instance, heat stress was previously shown to induce the expression of *clpP1* and *clpP2*, resulting in an increased number of heterocomplexes, which might result in elevated proteolytic rates for removal of misfolded proteins.^16^ In addition, it is intriguing to speculate that metabolites containing long aliphatic chains may act as native stimulants. While the existence of such metabolites has to be investigated in future studies, it is important to note that activating peptides have previously been reported for heterooligomeric MtClpPs.^17,19^ In fact, these small molecules bind to active sites and thereby enhance activity.^18–20^

In total, our in-depth analysis of LmClpP1/2 activity revealed two principles of proteolytic stimulation that both rely on elevated affinity between LmClpPs and LmClpX. This phenomenon appears to be special to the *Listeria* system, which is already unique through its unusual expression of two ClpP isoforms. Due to the fundamental relevance of ClpP for cell homeostasis under stress conditions, both activation pathways ensure a boost in ClpP activity when needed, while remaining tightly regulated by ClpX interactions in order to prevent uncontrolled damage.

## References

[cit1] Katayama-Fujimura Y., Gottesman S., Maurizi M. R. (1987). J. Biol. Chem..

[cit2] Kress W., Maglica Ž., Weber-Ban E. (2009). Res. Microbiol..

[cit3] Sauer R. T., Baker T. A. (2011). Annu. Rev. Biochem..

[cit4] Baker T. A., Sauer R. T. (2012). Biochim. Biophys. Acta.

[cit5] Wang J., Hartling J. A., Flanagan J. M. (1997). Cell.

[cit6] Ortega J., Singh S. K., Ishikawa T., Maurizi M. R., Steven A. C. (2000). Mol. Cell.

[cit7] Thompson M. W., Maurizi M. R. (1994). J. Biol. Chem..

[cit8] Lee M. E., Baker T. A., Sauer R. T. (2010). J. Mol. Biol..

[cit9] Böttcher T., Sieber S. A. (2008). Angew. Chem., Int. Ed. Engl..

[cit10] Gersch M., Kolb R., Alte F., Groll M., Sieber S. A. (2014). J. Am. Chem. Soc..

[cit11] Hackl M. W., Lakemeyer M., Dahmen M., Glaser M., Pahl A., Lorenz-Baath K., Menzel T., Sievers S., Bottcher T., Antes I., Waldmann H., Sieber S. A. (2015). J. Am. Chem. Soc..

[cit12] Pahl A., Lakemeyer M., Vielberg M. T., Hackl M. W., Vomacka J., Korotkov V. S., Stein M. L., Fetzer C., Lorenz-Baath K., Richter K., Waldmann H., Groll M., Sieber S. A. (2015). Angew. Chem., Int. Ed. Engl..

[cit13] Martin A., Baker T. A., Sauer R. T. (2007). Mol. Cell.

[cit14] Zeiler E., Braun N., Böttcher T., Kastenmüller A., Weinkauf S., Sieber S. A. (2011). Angew. Chem., Int. Ed. Engl..

[cit15] Zeiler E., List A., Alte F., Gersch M., Wachtel R., Poreba M., Drag M., Groll M., Sieber S. A. (2013). Proc. Natl. Acad. Sci. U. S. A..

[cit16] Dahmen M., Vielberg M. T., Groll M., Sieber S. A. (2015). Angew. Chem., Int. Ed. Engl..

[cit17] Akopian T., Kandror O., Raju R. M., Unnikrishnan M., Rubin E. J., Goldberg A. L. (2012). EMBO J..

[cit18] Schmitz K. R., Carney D. W., Sello J. K., Sauer R. T. (2014). Proc. Natl. Acad. Sci. U. S. A..

[cit19] Li M., Kandror O., Akopian T., Dharkar P., Wlodawer A., Maurizi M. R., Goldberg A. L. (2016). J. Biol. Chem..

[cit20] Famulla K., Sass P., Malik I., Akopian T., Kandror O., Alber M., Hinzen B., Ruebsamen-Schaeff H., Kalscheuer R., Goldberg A. L., Brötz-Oesterhelt H. (2016). Mol. Microbiol..

[cit21] Schmitz K. R., Sauer R. T. (2014). Mol. Microbiol..

[cit22] Akopian T., Kandror O., Tsu C., Lai J. H., Wu W., Liu Y., Zhao P., Park A., Wolf L., Dick L. R., Rubin E. J., Bachovchin W., Goldberg A. L. (2015). J. Biol. Chem..

[cit23] Leodolter J., Warweg J., Weber-Ban E. (2015). PLoS One.

[cit24] Szyk A., Maurizi M. R. (2006). J. Struct. Biol..

[cit25] Gersch M., Stahl M., Poreba M., Dahmen M., Dziedzic A., Drag M., Sieber S. A. (2016). ACS Chem. Biol..

[cit26] Kato D., Boatright K. M., Berger A. B., Nazif T., Blum G., Ryan C., Chehade K. A., Salvesen G. S., Bogyo M. (2005). Nat. Chem. Biol..

[cit27] Kim Y. I., Burton R. E., Burton B. M., Sauer R. T., Baker T. A. (2000). Mol. Cell.

[cit28] Joshi S. A., Hersch G. L., Baker T. A., Sauer R. T. (2004). Nat. Struct. Mol. Biol..

[cit29] Krojer T., Sawa J., Schafer E., Saibil H. R., Ehrmann M., Clausen T. (2008). Nature.

[cit30] Brötz-Oesterhelt H., Beyer D., Kroll H. P., Endermann R., Ladel C., Schroeder W., Hinzen B., Raddatz S., Paulsen H., Henninger K., Bandow J. E., Sahl H. G., Labischinski H. (2005). Nat. Med..

[cit31] Lee B. G., Park E. Y., Lee K. E., Jeon H., Sung K. H., Paulsen H., Rübsamen-Schaeff H., Brötz-Oesterhelt H., Song H. K. (2010). Nat. Struct. Mol. Biol..

[cit32] Li D. H., Chung Y. S., Gloyd M., Joseph E., Ghirlando R., Wright G. D., Cheng Y. Q., Maurizi M. R., Guarne A., Ortega J. (2010). Chem. Biol..

[cit33] Gersch M., Famulla K., Dahmen M., Göbl C., Malik I., Richter K., Korotkov V. S., Sass P., Rübsamen-Schaeff H., Madl T., Brötz-Oesterhelt H., Sieber S. A. (2015). Nat. Commun..

[cit34] Carney D. W., Compton C. L., Schmitz K. R., Stevens J. P., Sauer R. T., Sello J. K. (2014). ChemBioChem.

