# Supplementary material for: Insights into ClpXP proteolysis: heterooligomerization and partial deactivation enhance chaperone affinity and substrate turnover in Listeria monocytogenes
Source: Chem Sci. 2016 Oct 28;8(2):1592–600. doi: 10.1039/c6sc03438a (PMC5361862; doi:10.1039/c6sc03438a)
Supplement: Supplementary file 1 [file SC-008-C6SC03438A-s001.pdf]

Electronic Supplementary Material (ESI) for Chemical Science.
This journal is © The Royal Society of Chemistry 2016

Insights into ClpXP proteolysis: Heterooligomerization and partial deactivation enhance chaperone affinity and substrate turnover in *Listeria monocytogenes*

Dóra Balogh[1,3], Maria Dahmen[1,3], Matthias Stahl[1], Marcin Poreba[2], Malte Gersch[1,4], Marcin Drag[2] and Stephan A. Sieber[*,1]*

[1] Center for Integrated Protein Science at the Department of Chemistry, Technische Universität München, Lichtenbergstrasse 4, Garching, D-85747, Germany.

[2] Department of Bioorganic Chemistry, Faculty of Chemistry, Wrocław University of Technology, Wybrzeże Wyspiańskiego 27, 50-370 Wrocław, Poland.

[3] D.B. and M.D. contributed equally to this study.

[4] Present address: Medical Research Council Laboratory of Molecular Biology, Francis Crick Avenue, Cambridge, CB2 0QH, UK

* Corresponding author: S.A.S., stephan.sieber@mytum.de

Supporting Information

1. Supplementary Figures....................S2

2. Supplementary Tables....................S5

3. Biochemical Procedures....................S11

4. Chemical Synthesis....................S15

5. HPLC Chromatograms....................S17

6. MS Spectra....................S18

7. Supplementary References....................S22

1. Supplementary Figures

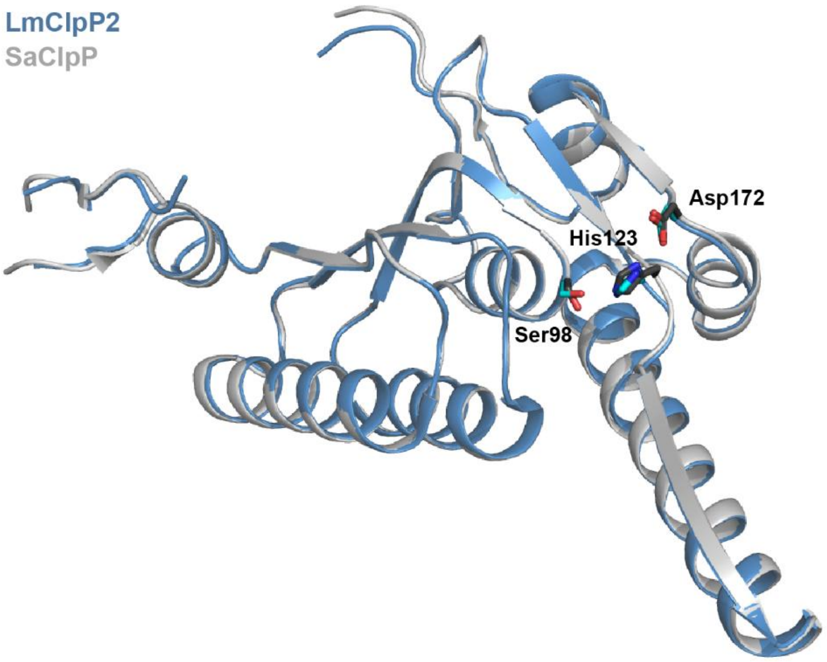

Fig. S1 Structural superposition of LmClpP2 from LmClpP1/2 heterocomplex (PDB code 4RYF, blue) and SaClpP (PDB code 3V5E, gray). Residues of the catalytic triad are shown in cyan for LmClpP2 and dark gray for SaClpP, heteroatoms O and N are shown in red and blue, respectively. The figure was made with PyMOL 1.3.[1]

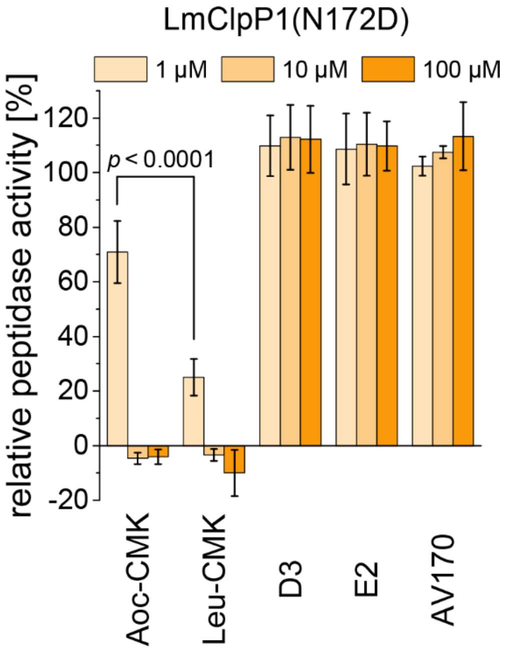

Fig. S2 Peptidase assay (1 µM LmClpP1(N172D) and 200 µM Ac-Ala-hArg-Leu-ACC substrate). Three different inhibitor concentrations were tested. Data are normalized to DMSO control as 100%. The dataset represents two independent experiments which were measured in triplicate (mean ± sd). *p*-value was determined by Student's *t*-test.

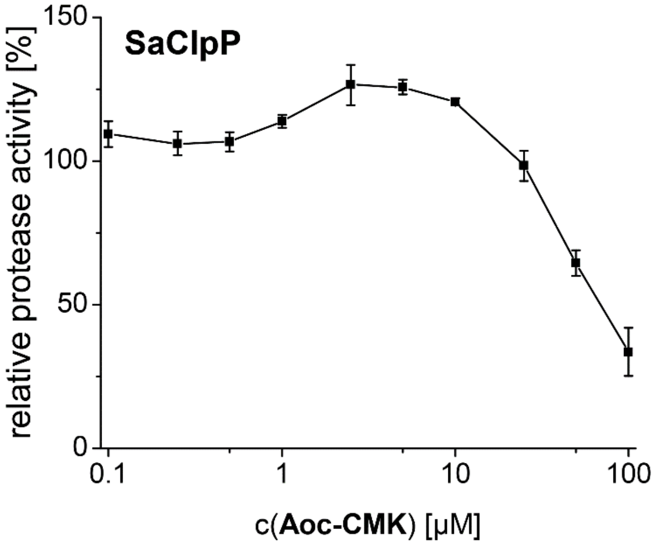

Fig. S3 Protease activity of SaClpP with **Aoc-CMK** (0.2 µM $SaClpP_{14}$, 0.4 µM $SaClpX_6$ and 0.4 µM eGFP-SsrA). Data represent two independent experiments which were measured in triplicate (mean ± sd).

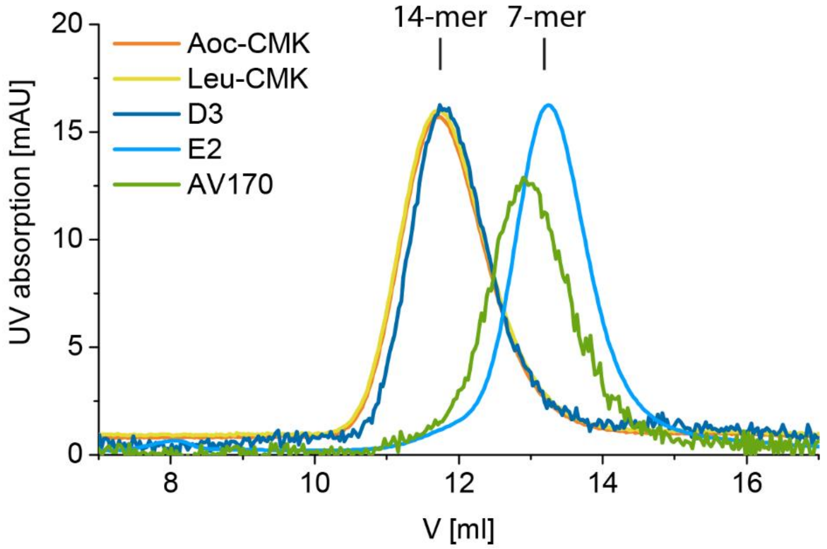

Fig. S4 Size-exclusion chromatograms of LmClpP2 treated with inhibitors. Treatment with **Aoc-CMK**, **Leu-CMK** and **D3** results in retention of the tetradecamer whereas **E2** and **AV170** induce de-oligomerization to heptamers.

2. Supplementary Tables

Table S1 Names and abbreviations of non-natural amino acids used in the peptidase substrate library screening.

Abbreviation	Name
D-Ala	D-alanine
D-Leu	D-leucine
D-Asp	D-aspartic acid
D-Arg	D-arginine
D-Phe	D-phenylalanine
Cit	citrulline
Orn	ornithine
hArg	homoarginine
Glu(O-Bzl)	glutamic acid 4-benzyl ester
Asp(O-Bzl)	aspartic acid 4-benzyl ester
hPhe	homophenylalanine
Cha	cyclohexylalanine
Thr(O-Bzl)	*O*-benzylthreonine
Phe(3,4-Cl2)	3,4-dichlorophenylalanine
Pip	piperidine-2-carboxylic acid
Abu	2-aminobutanoic acid
nVal	norvaline
Met(O2)	methionine sulfone
Dap	2,3-diaminopropionic acid
Dab	2,4-diaminobutyric acid
Phe(F5)	pentafluorophenylalanine
Bpa	*p*-benzoylphenylalanine
hLeu	homoleucine
nLeu	norleucine
Tic	1,2,3,4-tetrahydroisoquinoline-3-carboxylic acid
Igl	2-indanylglycine
2-Aoc	2-aminooctanoic acid
hSer	homoserine
Chg	cyclohexylglycine
dhLeu	dehydroleucine
Tle	*tert*-leucine
Arg(NO2)	N_ω-nitroarginine
Glu(O-Me)	glutamic acid γ-methyl ester
Asp(O-Me)	aspartic acid γ-methyl ester
Phe(4-Cl)	*p*-chlorophenylalanine
4-Pal	4-pyridylalanine
Ser(O-Bzl)	*O*-benzylserine
His(N-Bzl)	*N*(im)-benzylhistidine
Hnv	β-hydroxynorvaline

Hyp	4-hydroxyproline
Lys(TFA)	*N*-6-trifluoroacetyllysine

Table S2 Peptidase activity of LmClpP variants (1 µM) with the substrates of the P1 library (100 µM Ac-Ala-hArg-Xaa-ACC). Please refer to Table S1 for the names of non-natural amino acids. Data were measured in triplicates. [a]Peptide could not be obtained.

P1 amino acid	LmClpP2	LmClpP1^{m}/2^{wt}	LmClpP1^{wt}/2^{wt}	LmClpP1^{wt}/2^{m}
	peptidase activity (au ± sd)			
Ala	0.498 ± 0.010	0.343 ± 0.047	0.324 ± 0.007	0.022 ± 0.013
Arg	0.005 ± 0.017	-0.002 ± 0.034	0.000 ± 0.036	-0.021 ± 0.017
Asn	0.250 ± 0.017	0.131 ± 0.015	0.188 ± 0.019	0.019 ± 0.006
Asp	-0.028 ± 0.018	-0.017 ± 0.042	-0.013 ± 0.031	-0.066 ± 0.035
Gln	0.036 ± 0.011	0.020 ± 0.004	0.116 ± 0.010	0.090 ± 0.003
Glu	0.040 ± 0.015	0.007 ± 0.002	0.156 ± 0.013	0.141 ± 0.006
Gly	0.390 ± 0.003	0.214 ± 0.004	0.254 ± 0.046	0.012 ± 0.002
His	0.003 ± 0.012	0.002 ± 0.017	0.006 ± 0.009	0.011 ± 0.010
Ile	0.007 ± 0.010	0.004 ± 0.002	0.023 ± 0.007	0.003 ± 0.003
Leu	3.779 ± 0.014	3.783 ± 0.053	4.075 ± 0.044	0.457 ± 0.009
Lys	-0.001 ± 0.002	-0.031 ± 0.016	0.009 ± 0.080	-0.018 ± 0.025
Met	7.237 ± 0.103	9.334 ± 0.309	8.524 ± 0.221	0.403 ± 0.004
Phe	1.325 ± 0.049	0.868 ± 0.005	0.868 ± 0.017	0.035 ± 0.003
Pro	0.009 ± 0.004	-0.013 ± 0.010	0.006 ± 0.002	0.021 ± 0.002
Ser	0.054 ± 0.001	0.034 ± 0.036	0.038 ± 0.006	0.008 ± 0.019
Thr	-0.015 ± 0.003	-0.032 ± 0.012	-0.014 ± 0.019	-0.005 ± 0.003
Trp	0.066 ± 0.003	0.053 ± 0.003	0.060 ± 0.007	0.016 ± 0.006
Tyr	0.698 ± 0.053	0.463 ± 0.012	0.483 ± 0.025	0.012 ± 0.003
Val	0.048 ± 0.027	0.010 ± 0.007	0.031 ± 0.004	0.010 ± 0.005
D-Ala	0.025 ± 0.020	-0.013 ± 0.020	0.007 ± 0.002	0.007 ± 0.005
D-Leu	0.028 ± 0.004	0.015 ± 0.008	0.021 ± 0.000	-0.007 ± 0.041
D-Asp	0.015 ± 0.005	-0.002 ± 0.004	0.016 ± 0.005	0.017 ± 0.007
D-Arg	0.010 ± 0.003	0.002 ± 0.006	0.002 ± 0.001	0.012 ± 0.002
D-Phe	0.043 ± 0.036	0.022 ± 0.006	0.016 ± 0.007	0.015 ± 0.005
Cit	0.012 ± 0.129	0.063 ± 0.089	0.051 ± 0.012	0.015 ± 0.008
Orn	-0.327 ± 0.097	-0.155 ± 0.077	0.054 ± 0.101	0.011 ± 0.087
hArg	0.011 ± 0.003	0.003 ± 0.006	0.010 ± 0.006	0.012 ± 0.003
Glu(O-Bzl)	4.319 ± 0.167	4.779 ± 0.458	4.578 ± 0.220	0.000 ± 0.003
Asp(O-Bzl)	5.907 ± 0.033	4.053 ± 0.123	5.214 ± 0.092	0.049 ± 0.003
hPhe	2.932 ± 0.031	2.959 ± 0.037	2.658 ± 0.032	0.014 ± 0.001
Cha	0.818 ± 0.007	1.067 ± 0.028	0.937 ± 0.099	-0.023 ± 0.039
Thr(O-Bzl)	0.011 ± 0.001	0.019 ± 0.016	0.013 ± 0.002	0.017 ± 0.003
Phe(3,4-Cl2)	0.428 ± 0.034	0.197 ± 1.373	0.446 ± 0.438	-0.055 ± 0.020
Pip	n.d.[a]	n.d.[a]	n.d.[a]	n.d.[a]
Abu	0.226 ± 0.015	0.284 ± 0.079	0.291 ± 0.020	0.037 ± 0.007

nVal	0.914 ± 0.022	1.161 ± 0.017	1.226 ± 0.066	0.166 ± 0.043
Met(O2)	1.496 ± 0.018	2.826 ± 0.043	2.467 ± 0.014	0.018 ± 0.002
Dap	0.030 ± 0.038	-0.043 ± 0.060	0.016 ± 0.018	0.103 ± 0.028
Dab	-0.009 ± 0.025	-0.046 ± 0.008	0.009 ± 0.016	0.045 ± 0.006
Phe(F5)	0.011 ± 0.002	0.014 ± 0.010	0.026 ± 0.008	0.002 ± 0.002
Bpa	0.557 ± 0.014	0.688 ± 0.055	0.600 ± 0.020	0.000 ± 0.020
hLeu	3.187 ± 0.024	7.057 ± 0.134	6.077 ± 0.220	0.033 ± 0.005
nLeu	2.710 ± 0.037	5.156 ± 0.153	4.533 ± 0.102	0.091 ± 0.002
Tic	0.100 ± 0.002	0.144 ± 0.003	0.153 ± 0.003	0.027 ± 0.003
Igl	0.013 ± 0.002	0.015 ± 0.003	0.030 ± 0.005	0.006 ± 0.001
2-Aoc	6.619 ± 0.078	28.009 ± 0.282	21.948 ± 0.426	0.071 ± 0.002
hSer	0.089 ± 0.014	0.058 ± 0.002	0.241 ± 0.015	0.180 ± 0.035
Chg	0.029 ± 0.004	0.016 ± 0.002	0.044 ± 0.002	0.017 ± 0.005
dhLeu	n.d.[a]	n.d.[a]	n.d.[a]	n.d.[a]
Tle	n.d.[a]	n.d.[a]	n.d.[a]	n.d.[a]
Arg(NO2)	0.018 ± 0.003	0.023 ± 0.004	0.033 ± 0.006	0.010 ± 0.008
Glu(O-Me)	1.429 ± 0.014	2.116 ± 0.060	1.926 ± 0.027	0.050 ± 0.005
Asp(O-Me)	1.012 ± 0.022	0.929 ± 0.043	1.053 ± 0.021	0.093 ± 0.001
Phe(4-Cl)	1.084 ± 0.019	1.577 ± 0.188	1.283 ± 0.041	0.128 ± 0.004
4-Pal	0.066 ± 0.007	0.068 ± 0.000	0.086 ± 0.012	0.028 ± 0.000
Ser(O-Bzl)	1.488 ± 0.016	2.274 ± 0.040	1.997 ± 0.043	0.064 ± 0.005
His(N-Bzl)	-0.001 ± 0.002	0.001 ± 0.004	0.012 ± 0.004	0.012 ± 0.009
Hnv	0.192 ± 0.013	0.276 ± 0.030	0.268 ± 0.010	0.123 ± 0.123
Hyp	-0.081 ± 0.016	-0.122 ± 0.090	0.018 ± 0.012	-0.005 ± 0.011
Lys(TFA)	0.524 ± 0.009	0.784 ± 0.023	0.710 ± 0.008	0.006 ± 0.007

Table S3 Peptidase activity of LmClpP variants (1 µM) with the substrates of the P2 library (100 µM Ac-Ala-Xaa-Leu-ACC). Please refer to Table S1 for the names of non-natural amino acids. Data were measured in triplicates. [a]Peptide could not be obtained.

P2 amino acid	LmClpP2	LmClpP1^{m}/2^{wt}	LmClpP1^{wt}/2^{wt}	LmClpP1^{wt}/2^{m}
	peptidase activity (au ± sd)			
Ala	0.118 ± 0.015	0.118 ± 0.019	0.044 ± 0.002	-0.002 ± 0.007
Arg	0.777 ± 0.022	0.819 ± 0.026	0.313 ± 0.010	0.037 ± 0.011
Asn	0.057 ± 0.002	0.052 ± 0.012	0.022 ± 0.002	0.003 ± 0.002
Asp	0.002 ± 0.003	-0.015 ± 0.008	-0.010 ± 0.002	-0.003 ± 0.001
Gln	0.391 ± 0.009	0.374 ± 0.015	0.160 ± 0.009	0.020 ± 0.007
Glu	0.030 ± 0.004	0.017 ± 0.003	0.002 ± 0.001	0.004 ± 0.004
Gly	0.017 ± 0.002	0.022 ± 0.031	0.001 ± 0.003	0.007 ± 0.006
His	0.099 ± 0.004	0.075 ± 0.007	0.037 ± 0.004	0.001 ± 0.007
Ile	0.049 ± 0.002	0.028 ± 0.006	0.022 ± 0.004	0.015 ± 0.004
Leu	0.371 ± 0.025	0.203 ± 0.031	0.132 ± 0.018	0.025 ± 0.013
Lys	0.618 ± 0.003	0.820 ± 0.002	0.294 ± 0.007	0.045 ± 0.001
Met	0.288 ± 0.011	0.407 ± 0.056	0.198 ± 0.014	0.046 ± 0.003
Phe	0.080 ± 0.056	0.011 ± 0.004	0.010 ± 0.002	0.002 ± 0.002

Pro	-0.002 ± 0.005	-0.013 ± 0.002	-0.008 ± 0.003	0.008 ± 0.002
Ser	0.302 ± 0.003	0.264 ± 0.002	0.105 ± 0.001	0.014 ± 0.002
Thr	0.432 ± 0.021	0.352 ± 0.023	0.151 ± 0.011	0.029 ± 0.007
Trp	0.324 ± 0.024	0.065 ± 0.001	0.092 ± 0.012	-0.003 ± 0.014
Tyr	-0.001 ± 0.018	0.002 ± 0.001	-0.024 ± 0.014	-0.010 ± 0.017
Val	0.012 ± 0.013	0.001 ± 0.001	-0.003 ± 0.004	0.000 ± 0.003
D-Ala	-0.002 ± 0.002	-0.012 ± 0.001	0.000 ± 0.019	-0.002 ± 0.000
D-Leu	0.006 ± 0.003	0.002 ± 0.005	-0.008 ± 0.011	0.002 ± 0.002
D-Asp	-0.001 ± 0.003	-0.027 ± 0.026	0.002 ± 0.009	0.008 ± 0.005
D-Arg	n.d.[a]	n.d.[a]	n.d.[a]	n.d.[a]
D-Phe	-0.115 ± 0.117	-0.001 ± 0.034	-0.029 ± 0.016	0.001 ± 0.007
Cit	0.427 ± 0.022	0.428 ± 0.002	0.183 ± 0.024	0.020 ± 0.003
Orn	0.290 ± 0.002	0.308 ± 0.011	0.134 ± 0.005	0.033 ± 0.003
hArg	1.205 ± 0.016	1.645 ± 0.022	0.592 ± 0.011	0.149 ± 0.007
Glu(O-Bzl)	0.219 ± 0.021	0.201 ± 0.015	0.095 ± 0.010	0.003 ± 0.010
Asp(O-Bzl)	0.002 ± 0.005	-0.016 ± 0.004	-0.010 ± 0.002	0.003 ± 0.002
hPhe	0.051 ± 0.004	0.109 ± 0.003	0.034 ± 0.004	0.025 ± 0.005
Cha	0.305 ± 0.021	0.148 ± 0.011	0.019 ± 0.008	0.009 ± 0.016
Thr(O-Bzl)	0.037 ± 0.002	0.031 ± 0.016	-0.007 ± 0.009	0.015 ± 0.008
Phe(3,4-Cl2)	0.050 ± 0.010	0.025 ± 0.005	0.000 ± 0.003	0.007 ± 0.002
Pip	0.001 ± 0.014	-0.018 ± 0.013	-0.006 ± 0.021	-0.003 ± 0.002
Abu	0.030 ± 0.022	0.000 ± 0.001	-0.009 ± 0.014	0.001 ± 0.003
nVal	0.419 ± 0.038	0.546 ± 0.047	0.035 ± 0.008	-0.005 ± 0.018
Met(O2)	0.475 ± 0.044	0.672 ± 0.049	0.056 ± 0.012	0.081 ± 0.014
Dap	-0.039 ± 0.124	0.018 ± 0.003	-0.006 ± 0.018	0.011 ± 0.001
Dab	0.105 ± 0.005	0.073 ± 0.003	0.006 ± 0.002	0.011 ± 0.001
Phe(F5)	0.037 ± 0.008	0.047 ± 0.002	-0.009 ± 0.011	0.008 ± 0.005
Bpa	0.017 ± 0.022	0.015 ± 0.015	-0.006 ± 0.002	0.013 ± 0.008
hLeu	0.353 ± 0.022	0.505 ± 0.190	0.050 ± 0.004	0.020 ± 0.026
nLeu	0.197 ± 0.002	0.322 ± 0.080	0.040 ± 0.008	0.030 ± 0.013
Tic	-0.019 ± 0.033	-0.007 ± 0.005	0.010 ± 0.014	0.018 ± 0.003
Igl	0.119 ± 0.004	0.096 ± 0.002	0.025 ± 0.029	0.008 ± 0.001
2-Aoc	0.049 ± 0.008	0.056 ± 0.076	0.012 ± 0.020	0.035 ± 0.063
hSer	0.244 ± 0.019	0.213 ± 0.018	0.021 ± 0.001	0.012 ± 0.010
Chg	0.070 ± 0.010	0.048 ± 0.007	0.001 ± 0.005	-0.011 ± 0.035
dhLeu	n.d.[a]	n.d.[a]	n.d.[a]	n.d.[a]
Tle	-0.001 ± 0.004	-0.010 ± 0.002	-0.013 ± 0.001	-0.013 ± 0.037
Arg(NO2)	1.118 ± 0.012	1.019 ± 0.048	0.123 ± 0.003	0.058 ± 0.010
Glu(O-Me)	0.382 ± 0.033	0.633 ± 0.019	0.042 ± 0.007	0.050 ± 0.007
Asp(O-Me)	0.014 ± 0.002	0.001 ± 0.002	0.000 ± 0.004	0.006 ± 0.001
Phe(4-Cl)	0.006 ± 0.025	0.007 ± 0.001	-0.006 ± 0.010	-0.001 ± 0.007
4-Pal	0.350 ± 0.004	0.272 ± 0.007	0.043 ± 0.007	0.027 ± 0.009
Ser(O-Bzl)	0.230 ± 0.020	0.233 ± 0.017	0.025 ± 0.001	0.024 ± 0.002
His(N-Bzl)	0.166 ± 0.022	0.124 ± 0.022	0.013 ± 0.006	0.035 ± 0.001
Hnv	0.321 ± 0.017	0.321 ± 0.036	0.025 ± 0.005	0.019 ± 0.002

Hyp	-0.003 ± 0.002	-0.008 ± 0.006	-0.012 ± 0.003	-0.011 ± 0.010
Lys(TFA)	1.068 ± 0.019	2.135 ± 0.040	0.141 ± 0.007	0.052 ± 0.007

Table S4 Peptidase activity of LmClpP variants (1 µM) with the substrates of the P3 library (100 µM Ac-Xaa-hArg-Leu-ACC). Please refer to Table S1 for the names of non-natural amino acids. Data were measured in triplicates. [a]Peptide could not be obtained.

P3 amino acid	LmClpP2	LmClpP1^{m}/2^{wt}	LmClpP1^{wt}/2^{wt}	LmClpP1^{wt}/2^{m}
	peptidase activity (au ± sd)			
Ala	1.073 ± 0.091	1.721 ± 0.145	1.629 ± 0.133	0.107 ± 0.007
Arg	-0.056 ± 0.008	-0.136 ± 0.017	-0.071 ± 0.006	-0.143 ± 0.002
Asn	0.222 ± 0.014	0.072 ± 0.032	0.247 ± 0.032	0.042 ± 0.009
Asp	-0.068 ± 0.027	-0.175 ± 0.003	-0.103 ± 0.061	-0.184 ± 0.008
Gln	0.080 ± 0.026	-0.016 ± 0.015	0.011 ± 0.068	-0.094 ± 0.018
Glu	0.089 ± 0.029	-0.095 ± 0.007	-0.022 ± 0.006	-0.137 ± 0.016
Gly	0.138 ± 0.013	0.031 ± 0.014	0.102 ± 0.020	-0.074 ± 0.010
His	0.378 ± 0.072	0.202 ± 0.020	0.478 ± 0.016	0.126 ± 0.020
Ile	0.003 ± 0.005	3.677 ± 0.201	3.090 ± 0.075	0.387 ± 0.025
Leu	0.005 ± 0.006	2.910 ± 0.291	2.336 ± 0.170	0.108 ± 0.016
Lys	-0.011 ± 0.020	-0.122 ± 0.007	-0.063 ± 0.017	-0.050 ± 0.020
Met	0.001 ± 0.001	1.179 ± 0.055	1.256 ± 0.016	0.155 ± 0.038
Phe	2.663 ± 0.089	4.251 ± 0.056	4.561 ± 0.126	1.497 ± 0.066
Pro	1.382 ± 0.216	3.397 ± 0.168	2.682 ± 0.163	-0.060 ± 0.039
Ser	0.364 ± 0.074	0.223 ± 0.029	0.297 ± 0.002	0.033 ± 0.023
Thr	0.528 ± 0.023	0.485 ± 0.010	0.583 ± 0.020	0.116 ± 0.011
Trp	1.290 ± 0.115	1.189 ± 0.164	2.124 ± 0.156	2.068 ± 0.013
Tyr	0.852 ± 0.053	0.633 ± 0.041	1.782 ± 0.038	1.665 ± 0.018
Val	1.411 ± 0.106	1.240 ± 0.081	1.082 ± 0.038	0.181 ± 0.070
D-Ala	0.066 ± 0.012	-0.083 ± 0.029	-0.030 ± 0.013	-0.156 ± 0.030
D-Leu	-0.007 ± 0.018	-0.099 ± 0.005	-0.070 ± 0.006	-0.128 ± 0.008
D-Asp	-0.063 ± 0.009	-0.210 ± 0.031	-0.118 ± 0.024	-0.216 ± 0.034
D-Arg	0.102 ± 0.002	0.033 ± 0.002	0.041 ± 0.002	-0.008 ± 0.003
D-Phe	0.315 ± 0.026	0.168 ± 0.020	0.147 ± 0.007	0.002 ± 0.005
Cit	0.066 ± 0.044	-0.127 ± 0.014	0.238 ± 0.015	0.191 ± 0.014
Orn	-0.024 ± 0.023	-0.097 ± 0.013	-0.044 ± 0.007	-0.038 ± 0.005
hArg	-0.022 ± 0.025	-0.183 ± 0.009	-0.045 ± 0.059	-0.126 ± 0.049
Glu(O-Bzl)	0.170 ± 0.011	0.115 ± 0.012	0.135 ± 0.007	0.027 ± 0.006
Asp(O-Bzl)	0.253 ± 0.037	0.152 ± 0.011	0.294 ± 0.024	0.170 ± 0.010
hPhe	0.442 ± 0.121	0.109 ± 0.104	1.392 ± 0.092	2.645 ± 0.035
Cha	0.661 ± 0.017	0.594 ± 0.004	0.957 ± 0.042	1.016 ± 0.023
Thr(O-Bzl)	1.057 ± 0.025	1.366 ± 0.020	1.083 ± 0.011	0.209 ± 0.012
Phe(3,4-Cl2)	3.912 ± 0.100	3.923 ± 0.132	4.219 ± 0.198	4.960 ± 0.129
Pip	n.d.[a]	n.d.[a]	n.d.[a]	n.d.[a]
Abu	1.196 ± 0.038	2.010 ± 0.046	1.722 ± 0.083	0.700 ± 0.031
nVal	2.088 ± 0.033	3.292 ± 0.035	2.518 ± 0.025	0.452 ± 0.006

Met(O2)	0.028 ± 0.006	-0.036 ± 0.021	-0.086 ± 0.017	-0.060 ± 0.007
Dap	-0.090 ± 0.021	-0.131 ± 0.033	-0.105 ± 0.043	-0.017 ± 0.035
Dab	-0.053 ± 0.018	-0.090 ± 0.008	-0.097 ± 0.012	-0.012 ± 0.017
Phe(F5)	0.097 ± 0.045	-0.075 ± 0.022	-0.084 ± 0.052	-0.027 ± 0.075
Bpa	0.057 ± 0.015	-0.030 ± 0.018	-0.028 ± 0.009	-0.044 ± 0.014
hLeu	0.815 ± 0.023	0.738 ± 0.009	0.629 ± 0.014	0.197 ± 0.002
nLeu	1.165 ± 0.041	1.244 ± 0.073	1.145 ± 0.023	0.416 ± 0.017
Tic	0.413 ± 0.015	0.323 ± 0.037	0.242 ± 0.018	0.002 ± 0.007
Igl	0.454 ± 0.031	0.260 ± 0.018	0.838 ± 0.037	1.697 ± 0.045
2-Aoc	2.527 ± 0.156	1.447 ± 0.028	5.529 ± 0.201	5.423 ± 0.313
hSer	0.219 ± 0.028	0.148 ± 0.039	0.157 ± 0.031	0.153 ± 0.024
Chg	1.736 ± 0.053	1.734 ± 0.056	1.401 ± 0.037	-0.030 ± 0.013
dhLeu	0.495 ± 0.016	0.435 ± 0.017	0.339 ± 0.006	0.022 ± 0.028
Tle	0.958 ± 0.013	1.017 ± 0.019	0.729 ± 0.007	0.026 ± 0.003
Arg(NO2)	-0.064 ± 0.019	-0.086 ± 0.017	-0.107 ± 0.024	-0.035 ± 0.020
Glu(O-Me)	0.249 ± 0.028	0.235 ± 0.021	0.305 ± 0.021	0.214 ± 0.008
Asp(O-Me)	0.118 ± 0.035	0.074 ± 0.013	0.205 ± 0.017	0.256 ± 0.020
Phe(4-Cl)	1.620 ± 0.033	1.625 ± 0.041	2.380 ± 0.046	1.981 ± 0.022
4-Pal	0.212 ± 0.003	0.134 ± 0.006	0.103 ± 0.004	0.025 ± 0.002
Ser(O-Bzl)	0.691 ± 0.077	0.663 ± 0.018	1.126 ± 0.019	1.230 ± 0.040
His(N-Bzl)	-0.041 ± 0.020	-0.083 ± 0.014	-0.099 ± 0.011	-0.047 ± 0.011
Hnv	0.111 ± 0.023	0.015 ± 0.004	-0.006 ± 0.009	0.048 ± 0.031
Hyp	1.152 ± 0.067	1.577 ± 0.015	1.075 ± 0.008	0.099 ± 0.003
Lys(TFA)	-0.091 ± 0.029	-0.001 ± 0.027	-0.031 ± 0.013	0.190 ± 0.016

Table S5 List of primers used in this study

Primer	Sequence (5’-3’)
LmGlyA_for	ggggacaagtttgtacaaaaaagcaggctttgtctatttacaaaagcaagataaggaag
LmGlyA_rev	ggggaccactttgtacaagaaagctgggtgttattaggcagcgaaagctaggttttgtttttctttgcctgc<br>gctgcctaaacttggataaagcggatattcatttg
LmPncB_for	ggggacaagtttgtacaaaaaagcaggctttacaaatttatttcaagatgatagtc
LmPncB_rev	ggggaccactttgtacaagaaagctgggtgttattaggcagcgaaagctaggttttgtttttctttgcctgc<br>gctgccaaacggcatatctagttcaac

.

3. Biochemical Procedures

Cloning, protein overexpression

LmClpP2, LmClpP2(S98A), LmClpP1(N172D) and SaClpP were obtained as described previously.[2-4] Expression constructs with C-terminal Strep-tag II were cloned in pET301 plasmids, overexpressed in *E. coli* BL21(DE3) and purified with affinity chromatography and gel filtration.

LmClpP1^{wt}/2^{wt}, LmClpP1^{wt}/2^{m}, LmClpP1^{m}/2^{wt} and LmClpP1^{wt}/2^{wt} heterocomplex variants were overexpressed and purified as detailed previously.[4] In short, C-terminally Strep-II-tagged LmClpP1 and C-terminally His$_6$-tagged LmClpP2 were introduced into pETDuet-1 vector. Proteins were overexpressed in *E. coli* BL21(DE3) at 37 °C for 6 h after induction with 1 mM isopropyl-β-D-thiogalactoside (IPTG). After harvest the cells were lysed in His-lysis-buffer (20 mM MOPS, 100 mM KCl, 1% CHAPS, 5% glycerol, pH 8.0). The proteins from the cleared cell lysate were captured by Ni^{2+} affinity chromatography in His buffers (20 mM MOPS, 100 mM KCl, 5% glycerol, pH 8.0, +40 mM imidazole for washing, +300 mM imidazole for elution) and a subsequent StrepTactin chromatography step in Strep buffers (100 mM Tris, 150 mM NaCl, 1 mM EDTA, pH 8.0, +2.5 mM desthiobiotin for elution). A final gel filtration was performed in LmClpP-GF buffer (20 mM MOPS, 100 mM KCl, 5% glycerol, pH 7.0).

Tagfree LmClpX was overexpressed in *E. coli* BL21(DE3) or in SG1146a (Δ*clpP*). In both cases, an expression construct equipped with an N-terminal His$_6$-tag and a TEV cleavage site in pET300 vector was used.[4] 4 L LB medium were inoculated (1:100) and grown to OD_{600} 0.6 at 37 °C. After induction with 0.5 mM IPTG the cells were incubated over night at 25 °C. The cells were harvested by centrifugation, washed in PBS, resuspended in ClpX lysis buffer (25 mM HEPES, 200 mM KCl, 1 mM DTT, 5% glycerol, 0.5 mM ATP, 5 mM $MgCl_2$, pH 7.6) and lysed by ultrasonication. The cell debris was removed by centrifugation (38,000 g, 40 min, 4 °C) and the cell lysate was loaded on a 5 mL HisTrap HP column (GE Healthcare) using an ÄKTA Purifier 10 system (GE Healthcare). The column was washed with 12 column volumes (CV) ClpX wash buffer (25 mM HEPES, 200 mM KCl, 1 mM DTT, 5% glycerol, 40 mM imidazole, pH 7.6). The protein was eluted with 6 CV ClpX elution buffer (25 mM HEPES, 200 mM KCl, 1 mM DTT, 5% glycerol, 300 mM imidazol, pH 7.6). The protein fractions were pooled, 1 mM EDTA and 1.25 mg TEV protease were added and the reaction mixture was incubated at 10 °C over night. The completeness of the TEV cleavage was verified by intact-protein mass-spectrometry. The protein solution was loaded on a Superdex 200 pg 16/60 column (GE Healthcare) and eluted in ClpX lysis buffer.

Tagfree SaClpX was purified as described previously.[5] In short, for the overexpression of SaClpX with an N-terminal His$_6$-tag and TEV site pET301 vector was used in *E. coli* BL21(DE3) cells. The cell lysate was loaded on a HisTrap HP column (GE Healthcare). TEV protease and 1 mM EDTA were added to the pooled fractions. After cleavage and removal of imidazole, the protein solution was loaded on a HisTrap HP column and the flow-through was collected which was further purified by gel filtration.

N-terminally Strep-II-tagged eGFP with a C-terminal SsrA tag (AGKEKQNLAFAA for *L. monocytogenes* and AANDENYALAA for *E. coli*) was overexpressed in *E. coli* KY2266 (Δ*clpXP*, Δ*lon*, Δ*hslVU*)[6] using pDEST007 expression vector and purified by affinity chromatography and gel filtration as described previously.[4, 5]

GlyA (UniProt entry Q8Y4B2) and PncB (UniProt entry Q8Y826) with an N-terminal Strep-II tag and a C-terminal LmSsrA tag (AGKEKQNLAFAA) were constructed from *L. monocytogenes* EGD-e genomic DNA

in pDEST007 plasmid vector with Gateway® Technology using the primers listed in Table S5. The plasmids were transformed into *E. coli* SG1146a cells. 2 L LB culture was induced with 0.2 µg/mL anhydroteracycline after reaching an OD_{600} of 0.6. GlyA was incubated at 37 °C for 5 hours and PncB at 25 °C over night. Cells were harvested by centrifugation, washed with PBS, resuspended in PBS and ultrasonicated on ice. The cell lysate was cleared by centrifugation (38,000 g, 40 min, 4 °C) and loaded on a pre-equlibrated 5 mL StrepTrap HP column (GE Healthcare). The column was washed with 6 CV binding buffer (100 mM Tris-HCl, 150 mM NaCl, 1 mM EDTA, pH 8.0) and eluted with 5 CV binding buffer + 2.5 mM desthiobiotin. EcRpoS and SaGudB with SsrA tags (AANDENYALAA and AGKSNNNFAVAA, respectively) were purified similarly as described elsewhere.[7]

Creatine kinase (10 127 566 001), lactate dehydrogenase (10 128 155 001) and pyruvate kinase (10 127 876 001) were purchased from Roche.

Peptidase assay

In the substrate library screening following fluorogenic tripeptide substrate libraries were used: Ac-Ala-hArg-Xaa-ACC for P1 site, Ac-Ala-Xaa-Leu-ACC for P2 site and Ac-Xaa-hArg-Leu-ACC for P3 site. 1 µL substrate (100x stock in DMSO, 100 µM final concentration) was added to a flat bottom black 96-well plate and equilibrated to 32 °C. LmClpP (1 µM) in preheated peptidase buffer (100 mM HEPES, 100 mM KCl, 15% glycerol, pH 7.0) was added to a final volume of 100 µL to start the reaction, and fluorescence (excitation: 380 nm, emission: 440 nm) was measured with an infinite M200Pro plate reader (Tecan). Data were recorded in triplicate.

For the testing of inhibitors 1 µL inhibitor (100x stock in DMSO, 1 µM, 10 µM and 100 µM final concentrations) and LmClpP (1 µM) in peptidase buffer (100 µL final volume) were incubated for 30 min at 32 °C. 1 µL (100x stock in DMSO, 200 µM final concentration) Ac-Ala-hArg-Leu-ACC substrate was added and the fluorescece was measured (380 nm, 430 nm). Data were recorded in triplicate and two independent experiments were performed.

Peptidase activity was determined by linear regression using Microsoft Excel and plots were made with Microcal OriginPro 2016.

Protease assay

Protease assays were carried out in flat bottom white 96-well plates in a final volume of 60 µL. 0.6 µL inhibitor (100x DMSO stock, 0.1 – 100 µM final concentrations), $ClpP_{14}$ (0.2 µM), $ClpX_6$ (0.4 µM) and ATP regeneration mix (4 mM ATP, 16 mM creatine phosphate, 20 U/mL creatine kinase) were pre-incubated for 15 min at 30 °C in protease buffer (25 mM HEPES, 200 mM KCl, 5 mM $MgCl_2$, 1 mM DTT, 10% glycerol, pH 7.6). 0.4 µM eGFP-SsrA substrate was added and fluorescence was measured (485 nm, 535 nm). Data were recorded in triplicate and at least two independent experiments were performed. Protease activity was determined by linear regression using Microsoft Excel and plots were made with Microcal OriginPro 2016.

For the determination of K_{app} values the $LmClpP_{14}$ concentration was varied (2 nM – 8000 nM). If needed, **Aoc-CMK** (100x stock in DMSO, final concentration = 25×$LmClpP_{14}$ concentration) or DMSO was added. In case of the GFP unfolding assay, LmClpP2(S98A) and LmClpP1(S98A)/LmClpP2(S98A)

mutants were used in varying concentrations (10 nM – 30 µM) in presence of 0.4 µM $LmClpX_6$ and 0.125 µM eGFP-SsrA. Data were recorded in triplicate and at least two independent experiments were performed. Slopes were determined by linear regression using Microsoft Excel. Protease activity was plotted against $LmClpP_{14}$ concentration in Microcal OriginPro 2016 and was fitted to the Hill equation: $y = V_{min} + (V_{max} - V_{min}) \frac{x^n}{K_{app}^n + x^n}$.

ATPase assay

0.85 µL **Aoc-CMK** (100x DMSO stock, 4.25 µM final concentration) or DMSO were added to a flat bottom transparent 96-well plate. $LmClpX_6$ (0.2 µM) and $LmClpP2_{14}$ (0.1 µM) were added in ATPase buffer (100 mM HEPES, 200 mM KCl, 20 mM $MgCl_2$, 1 mM DTT, 1 mM NADH, 2 mM phosphoenolpyruvate, 50 U/mL lactate dehydrogenase, 50 U/mL pyruvate kinase, 5% glycerol, pH 7.5) and incubated for 12 min at 37 °C. The reaction was started by the addition of 20 mM ATP. Absorption at 340 nm was measured. Three independent experiments with four replicates each were carried out. ATPase activity was determined by linear regression using Microsoft Excel after substraction of the background signal (measurement without LmClpX), the plot was made with Microcal OriginPro 2016.

Analysis of LmClpXP protein substrate digests

$LmClpP_{14}$ (0.2 µM), $LmClpX_6$ (0.4 µM), SsrA-tagged substrate protein (1 µM) and ATP regeneration mix (4 mM ATP, 16 mM creatine phosphate, 20 U/mL creatine kinase) were incubated over night at 37 °C in protease buffer (25 mM HEPES, 200 mM KCl, 5 mM $MgCl_2$, 1 mM DTT, 10% glycerol, pH 7.6).

The samples were desalted using reverse phase extraction cartridges (tC18 SepPak, 50 mg, Waters) on a vacuum manifold. Cartridges were washed three times with 1 mL acetonitrile (ACN), 1 mL 50% ACN with 0.5% formic acid (FA) and twice with 1 mL 0.1% FA. After addition of FA to a final concentration of 0.5% FA, the samples were loaded on the cartridges, washed with 1 mL 0.1% FA and twice with 1 mL 0.5% FA. Peptides were eluted into low binding reaction tubes (Eppendorf) with 3×200 µL 80% ACN with 0.5% FA. The eluates were vacuum dried. The dried samples were dissolved in 100 µL 1% FA, ultrasonicated for 15 min and filtered on a 0.45 µm pore size filter.

LC-MS/MS analysis was carried out on a Dionex UltiMate 3000 nano HPLC coupled to a Thermo Finnigan Orbitrap XL. Samples were loaded onto a C18 NanoTrap Column (Acclaim C18 PepMap100, 2 cm×10 µm i.d., 5 µm particle size, 300 Å pore size) and separated on a Dionex C18 PepMap RSLC (Acclaim C18 PepMap RSLC, 50 cm×75 µm i.d., 2 µm particle size, 100 Å pore size) column. Solvent A consisted of water + 0.1% FA + 5% DMSO and solvent B consisted of ACN + 0.1% FA + 5% DMSO. Separation was achieved at a constant flow rate of 0.2 µL/min using a gradient from 4% B to 30% B over 90 min and a subsequent wash-out to 80% B over 33 min. Full scans were carried out with an m/z range of 350 – 1400 at a resolution of 60000 followed by a TOP5 CID fragmentation step (35 eV collision energy, activation time: 30 ms) using dynamic exclusion (30 s).

Fragmentation spectra were searched using the SEQUEST HT algorithm against a custom compiled proteome including contaminants using Proteome Discoverer 1.4. Cleavages were allowed after every amino acid, but the search was limited to monoisotopic precursor ions and a peptide mass tolerance of < 10 ppm. Oxidation (+15.995 Da) was set as dynamic modification at methionine residues in all

runs. Peptides were validated by the Percolator algorithm with FDR = 0.01 and ΔCn = 0.05. All peptide-to-spectrum matches passing the validation were then exported and further analyzed in a custom-built and openly available web-script called Protein|Clpper (www.oc2.ch.tum.de). For a detailed depiction of the workflow see Gersch/Stahl/Poreba et al.[7]

Intact-protein mass-spectrometry

0.2 µM $LmClpP2_{14}$, 0.4 µM $LmClpP2_{6}$, and **Aoc-CMK** (100x DMSO stock, 1 µM, 5 µM, 10 µM and 100 µM final concentrations) were incubated with ATP regeneration mix (4 mM ATP, 16 mM creatine phosphate, 20 U/mL creatine kinase) in protease buffer (25 mM HEPES, 200 mM KCl, 5 mM $MgCl_2$, 1 mM DTT, 10% glycerol, pH 7.6) at 30 °C for 15 min. Measurements were carried out on a Dionex Ultimate 3000 HPLC system coupled to a Thermo LTQ-FT Ultra mass-spectrometer with electrospray ionisation source (spray voltage 4.0 kV, tube lens 110 V, capillary voltage 48 V, sheath gas 60 a.u., aux gas 10 a.u., sweep gas 0.2 a.u.). 5 µL of reaction mixtures were desalted with a Massprep desalting cartridge (Waters). The mass spectrometer was operated in positive mode collecting full scans at high resolution (R = 200,000) from m/z 600 to m/z 2000. Collected data was deconvoluted using the Thermo Xcalibur Xtract algorithm. Data was recorded in triplicate.

Analytical gel filtration

20 µM LmClpP2 was incubated for 10 min at room temperature with 100 µM inhibitor in GF buffer (20 mM MOPS, 100 mM KCl, 5% glycerol, pH 7.0). 150 µL of the samples were loaded on a pre-equilibrated Superdex 200 10/300 gel filtration column (GE Healthcare) connected to an ÄKTA Purifier 10 system (GE Healthcare) and eluted with 1 CV GF buffer. UV absorption was recorded at 280 nm. The oligomerization state was determined by comparison of the elution volumes to the calibration curve of the column (Gel Filtration Calibration Kit, GE Healthcare).

4. Chemical synthesis

CMK inhibitors

The general procedure for the peptide-CMK inhibitors was adapted fom Kato et al.[8] A 0.2 M solution of the Boc-*L*-Leu-OH amino acid (5 mmol) in anhydrous THF was stirred in a ice/acetone bath at -10 °C for 15 min. Then, 4-methylmorpholine (6.25 mmol, 1.25 eq) and isobutyl chloroformate (5.75 mmol, 1.15 eq) were added dropwise. The reaction was carried out in the ice/acetone bath for 45 min. In parallel, diazomethane (around 20 mmol) was generated according to the AL-180 (Aldrich Technical Bulletin) protocol. Next, the solution of mixed anhydrides was slowly added to the etheral solution of diazomethane at 0°C (ice bath). The mixture was stirred for 5 min, then the ice bath was removed and the reaction was carried out for 3 hours at room temperature. In order to obtain Boc-*L*-Leu-CH_2Cl, 15 mL of a 1:1 of concentrated HCl and glacial acetic acid was added dropwise to the mixture (room temperature). After Boc-*L*-Leu-CH_2N_2 was fully converted into Boc-*L*-Leu-CH_2Cl (HPLC analysis) the mixture was diluted 10x with ethyl acetate, transferred to a separatoty funnel and extracted with water (1x), saturated aqueous $NaHCO_3$ (3x) and brine (2x). The organic fraction was collected, dried over $MgSO_4$ and evaporated under reduced pressure. The Boc-*L*-Leu-CH_2Cl was obtained as a pale yellow oil. Next, the Boc group was removed with 25% solution of TFA in DCM (stirring for 30 min). After the deprotection reaction was complete (HPLC analysis), TFA and DCM were removed under reduced pressure and NH_2-*L*-Leu-CMK was obtained as pale yellow oil. The product was used for further synthesis without purification. In a separate synthesis the Ac-*L*-Phe(3,4-Cl_2)-*L*-hArg$(Boc)_2$-OH peptide fragment was synthesized on 2-chlorotrityl chloride resin. Fmoc-*L*-hArg$(Boc)_2$-OH (2 eq) was dissloved in the minimal volume of anhydrous DCM, followed by addition of DIPEA (3 eq). The amino acid was activated for a minute and poured onto the resin (1 eq). The reaction mixture was agitated for 5 hours. Then the Fmoc group was removed with 20% piperidine in dry DMF in three cycles (5 min, 5 min, 25 min). Next, the Fmoc-*L*-Phe(3,4-Cl_2)-OH (2 eq) amino acid was coupled to the NH_2-*L*-hArg$(Boc)_2$-resin (1 eq) using HATU (2 eq) and collidine (2 eq) as coupling reagents. The reaction was carried out for 3 hours. Next, the Fmoc group was removed as previously and the free N-termini was acetylated with AcOH (1.5 eq), HBTU (1.5 eq) and DIPEA (1.5 eq) for 30 min to obtain Ac-Phe(3,4-Cl_2)-*L*-hArg$(Boc)_2$-resin. After the acetylation was complete, the resin was washed with DMF (3x), DCM (3x), and MeOH (3 eq) and dried over P_2O_5 overnight. The peptide was cleaved from the resin with the use of DCM/TFE/AcOH (8:1:1, v/v/v) mixture (45 min). Then the peptide solution was filtered and solvents were evaporated under reduced pressure. The crude product was dissolved in acetonitrile/water (6:4, v/v) and lyophilized to obtain Ac-*L*-Phe(3,4-Cl_2)-*L*-hArg$(Boc)_2$-OH as a white powder. In the next step Ac-*L*-Phe(3,4-Cl_2)-*L*-hArg$(Boc)_2$-OH (1.1 eq) and HATU (1.1 eq) were dissolved in DMF, followed by the addition of collidine (2.2 eq). After 1 min of peptide activation the mixture was added into flask containing crude NH_2-*L*-Leu-CMK (1 eq). The coupling reaction was carried out for 45 min (HPLC analysis). Next, DMF was removed under reduced pressure (oil pump) and the 25% of TFA in DCM added in order to remove Boc groups from hArg (1 hour). Finally, the product (Ac-*L*-Phe(3,4-Cl_2)-*L*-hArg-*L*-Leu-CH_2Cl, **Leu-CMK**) was purified on semipreparative HPLC and lyophilized. The second inhibitor, Ac-*L*-Phe(3,4-Cl_2)-*L*-hArg-*L*-2-Aoc-CH_2Cl, **Aoc-CMK** was synthesized in a similar manner.

Both inhibitors were purified by HPLC on a Waters M600 solvent delivery module with a Waters M2489 detector system using a semi-preparative Waters Spherisorb S10ODS2 column (particle size 10 µm). The solvent composition was as follows: phase A (water/0.1% TFA) and phase B (ACN/0.1% TFA). The

purity of these compounds was confirmed by analytical HPLC system using a Waters Spherisorb S5ODS2 column (particle size 5 µm).

Characterization of **Leu-CMK**:
RP-HPLC (gradient: 100% phase A → 100% phase B in 40 min): t_R = 21.289 min
HRMS ESI calcd. for $C_{25}H_{37}Cl_3N_6O_4$ $[M+H]^+$: 591.2020, found 591.2040.

Characterization of **Aoc-CMK**:
RP-HPLC (gradient: 100% phase A → 100% phase B in 40 min): t_R = 21.971 min
HRMS ESI calcd. for $C_{27}H_{41}Cl_3N_6O_4$ $[M+H]^+$: 619.2333, found 619.2330.

5. HPLC Chromatograms

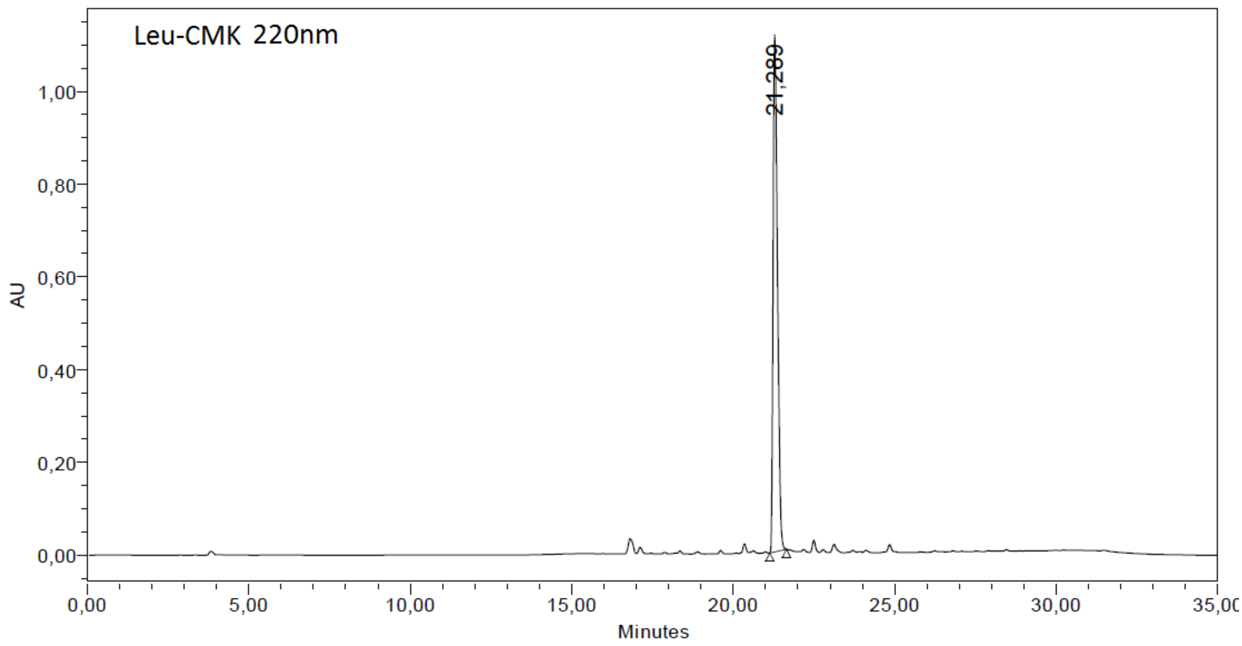

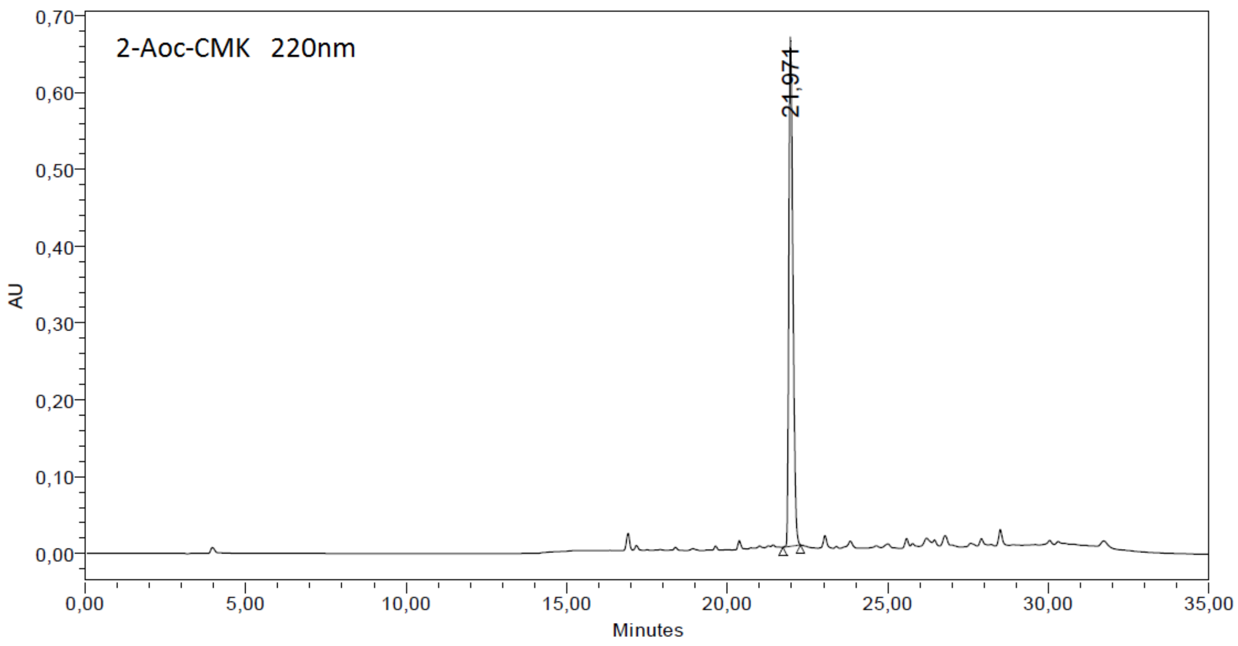

6. MS Spectra

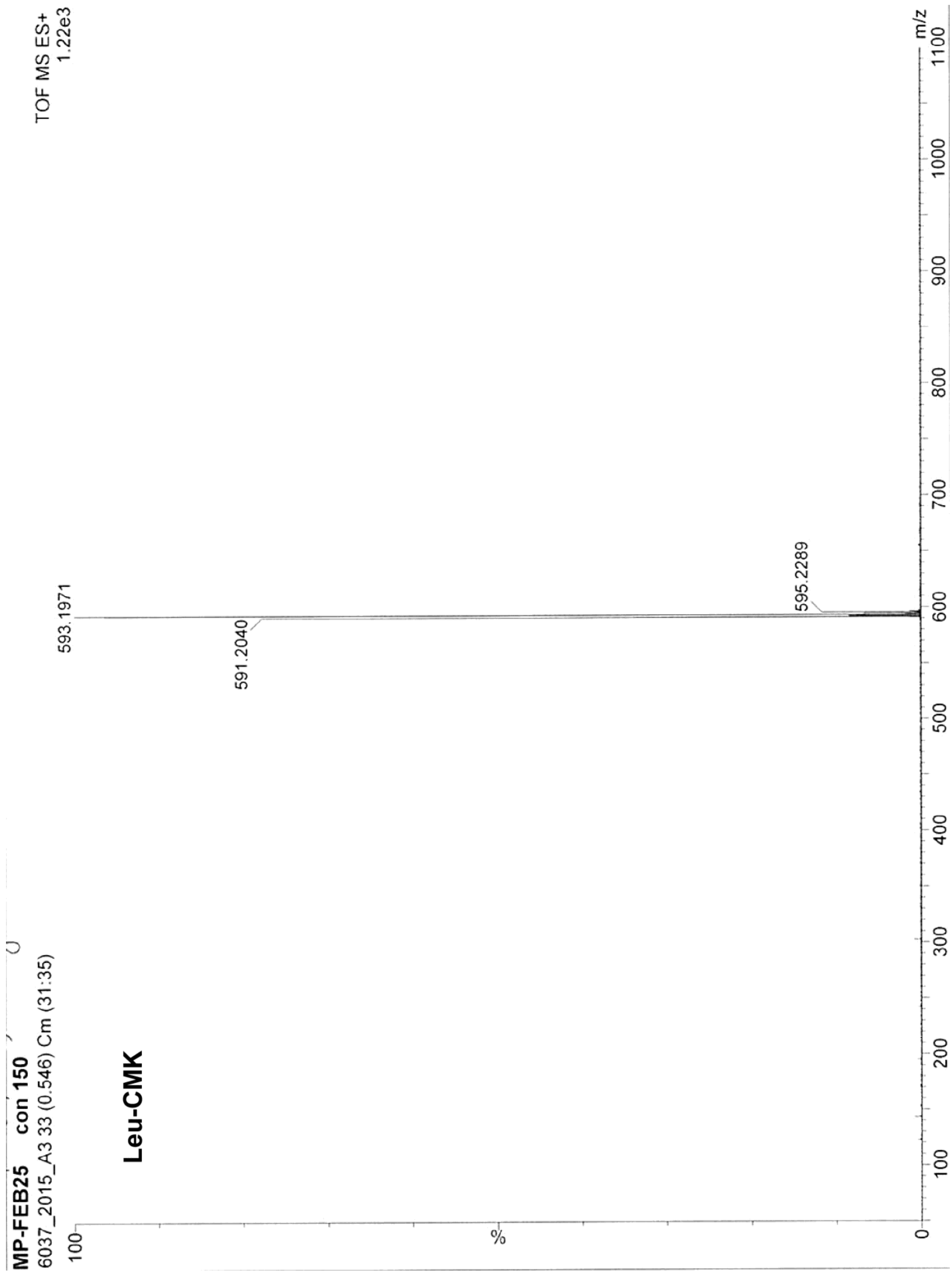

MP-FEB25 con 150
6037_2015_A3 33 (0.546) Cm (31:35)
TOF MS ES+
1.22e3
Leu-CMK
593.1971
591.2040
595.2289
%
100
0
m/z
100
200
300
400
500
600
700
800
900
1000
1100

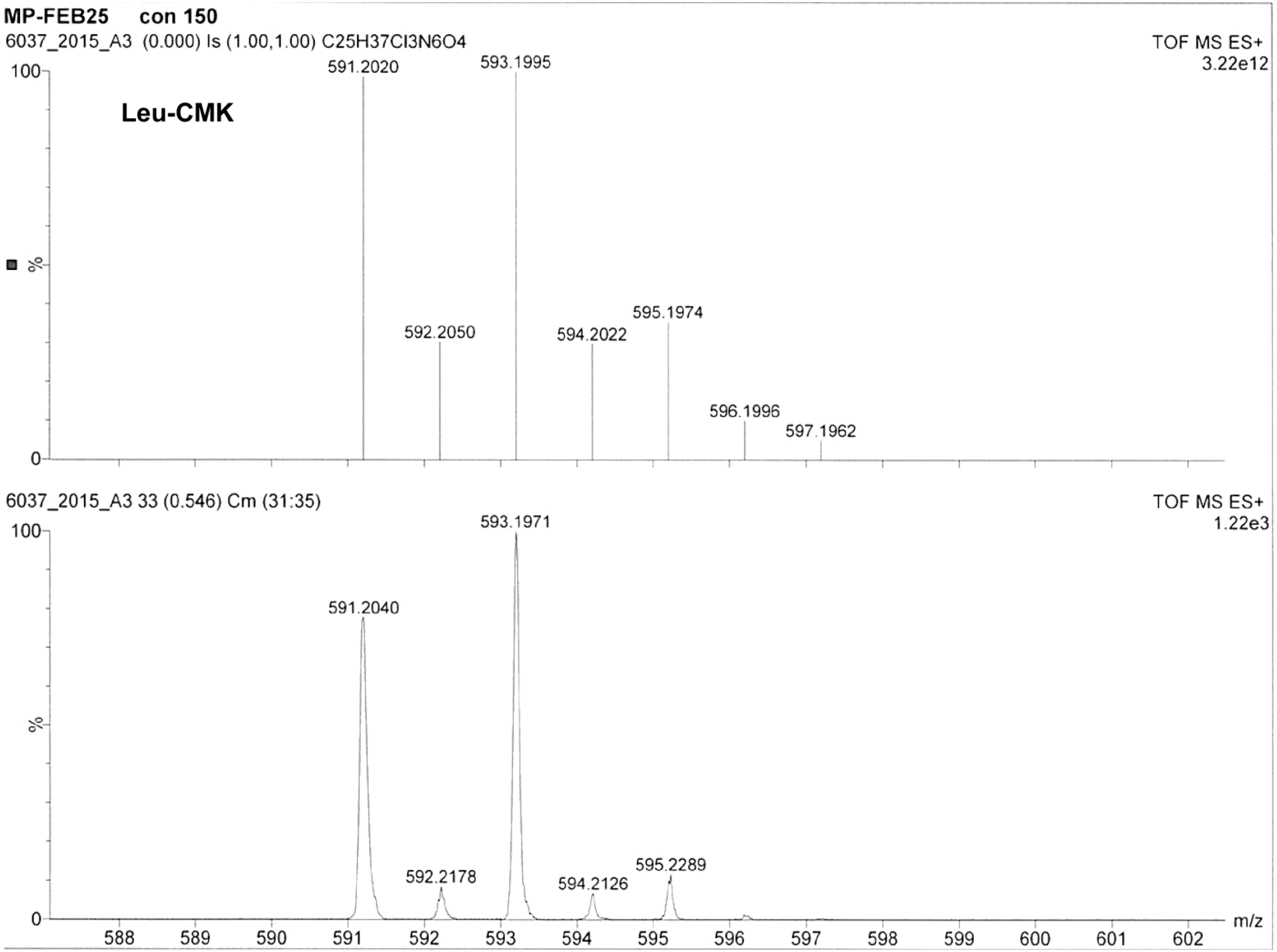
MP-FEB25 con 150
6037_2015_A3 (0.000) Is (1.00,1.00) C25H37Cl3N6O4
TOF MS ES+
3.22e12
Leu-CMK
591.2020
593.1995
592.2050
594.2022
595.1974
596.1996
597.1962
6037_2015_A3 33 (0.546) Cm (31:35)
TOF MS ES+
1.22e3
593.1971
591.2040
592.2178
594.2126
595.2289
100
%
0
588
589
590
591
592
593
594
595
596
597
598
599
600
601
602
m/z

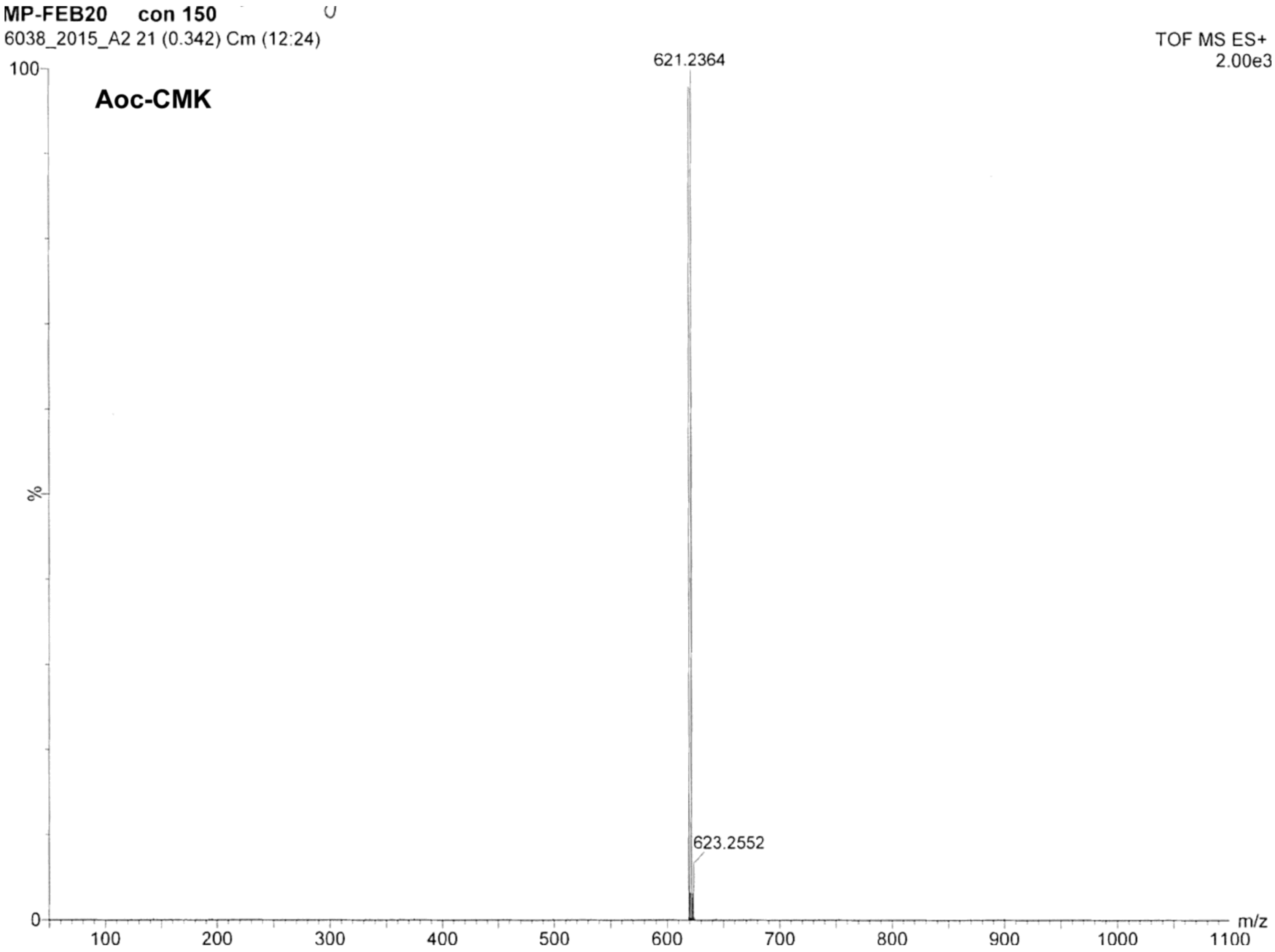
MP-FEB20 con 150
6038_2015_A2 21 (0.342) Cm (12:24)
TOF MS ES+
2.00e3
Aoc-CMK
621.2364
623.2552
100
%
0
100
200
300
400
500
600
700
800
900
1000
1100
m/z

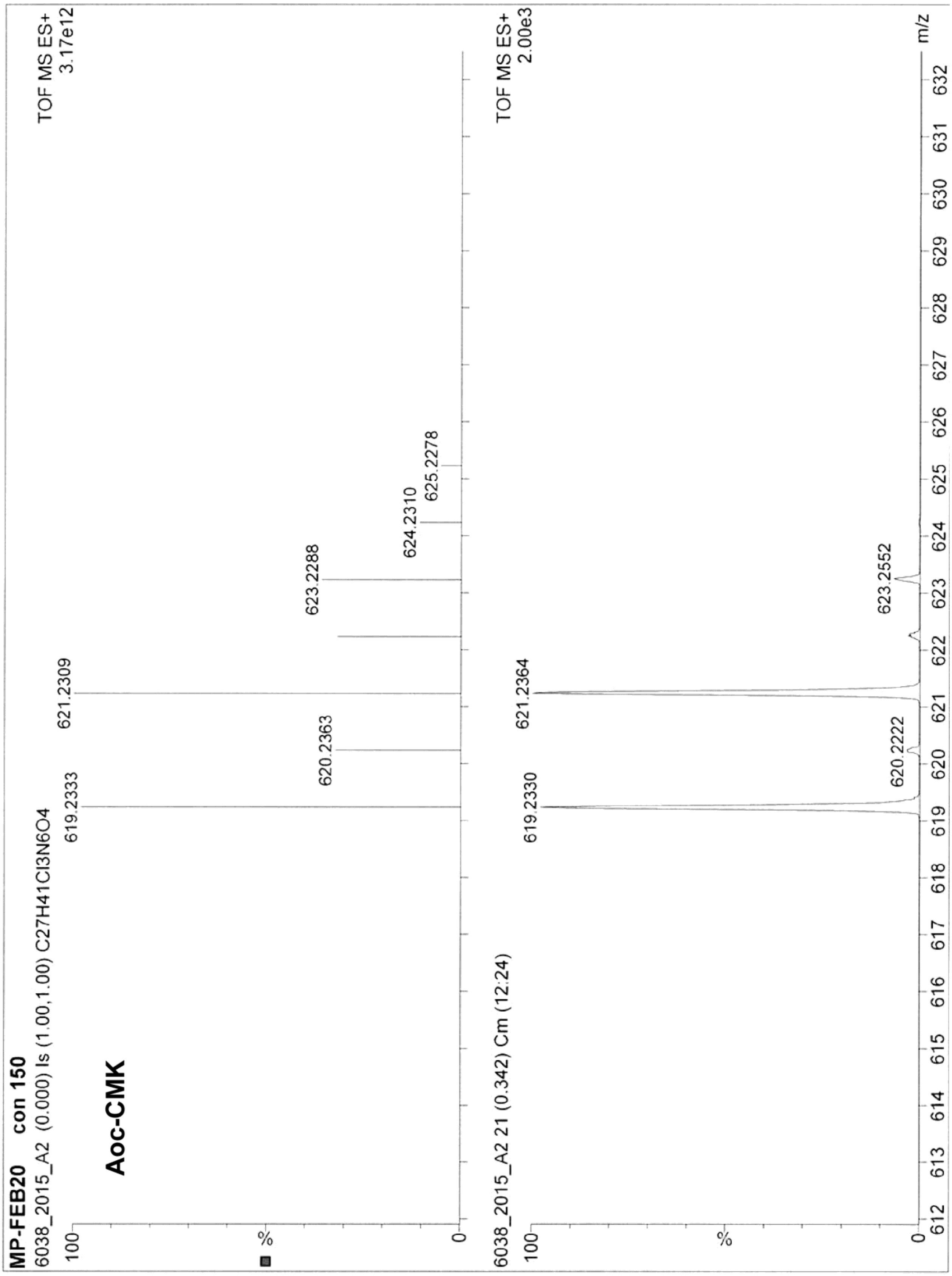
MP-FEB20 con 150
6038_2015_A2 (0.000) Is (1.00,1.00) C27H41Cl3N6O4
TOF MS ES+
3.17e12
Aoc-CMK
619.2333
620.2363
621.2309
623.2288
624.2310
625.2278
6038_2015_A2 21 (0.342) Cm (12:24)
TOF MS ES+
2.00e3
619.2330
620.2222
621.2364
623.2552
%
m/z

7. Supplementary references

1. The PyMOL Molecular Graphics System, Version 1.3 Schrodinger, LLC.
2. E. Zeiler, A. List, F. Alte, M. Gersch, R. Wachtel, M. Poreba, M. Drag, M. Groll and S. A. Sieber, *Proc. Natl. Acad. Sci. U. S. A.*, 2013, **110**, 11302-11307.
3. M. Gersch, A. List, M. Groll and S. A. Sieber, *J. Biol. Chem.*, 2012, **287**, 9484-9494.
4. M. Dahmen, M. T. Vielberg, M. Groll and S. A. Sieber, *Angew. Chem., Int. Ed. Engl.*, 2015, **54**, 3598-3602.
5. M. Gersch, K. Famulla, M. Dahmen, C. Gobl, I. Malik, K. Richter, V. S. Korotkov, P. Sass, H. Rubsamen-Schaeff, T. Madl, H. Brotz-Oesterhelt and S. A. Sieber, *Nat. Commun.,* 2015, **6**, 6320.
6. M. Kanemori, K. Nishihara, H. Yanagi and T. Yura, *J. Bacteriol.*, 1997, **179**, 7219-7225.
7. M. Gersch, M. Stahl, M. Poreba, M. Dahmen, A. Dziedzic, M. Drag and S. A. Sieber, *ACS Chem. Biol.*, 2016, **11**, 389-399.
8. D. Kato, K. M. Boatright, A. B. Berger, T. Nazif, G. Blum, C. Ryan, K. A. Chehade, G. S. Salvesen and M. Bogyo, *Nat. Chem. Biol.*, 2005, **1**, 33-38.
